# Tapping into the human spinal locomotor centres with transspinal stimulation

**DOI:** 10.1038/s41598-024-56579-0

**Published:** 2024-03-12

**Authors:** Andreas Skiadopoulos, Maria Knikou

**Affiliations:** 1 Klab4Recovery Research Program, The City University of New York, New York, USA; 2 Department of Physical Therapy, College of Staten Island, The City University of New York, Staten Island, NY USA; 3 PhD Program in Biology and Collaborative Neuroscience Program, Graduate Center of The City University of New York and College of Staten Island, New York, USA; 4 Klab4Recovery Research Program, Neurosciences/Graduate Center of CUNY, DPT Department/College of Staten Island, 2800 Victory Blvd, 5N-207, New York, 10314 USA

**Keywords:** Transspinal stimulation, Locomotor networks, Intralimb coordination, Interlimb coordination, Dynamic stability, Motor control, Central pattern generators, Translational research

## Abstract

Human locomotion is controlled by spinal neuronal networks of similar properties, function, and organization to those described in animals. Transspinal stimulation affects the spinal locomotor networks and is used to improve standing and walking ability in paralyzed people. However, the function of locomotor centers during transspinal stimulation at different frequencies and intensities is not known. Here, we document the 3D joint kinematics and spatiotemporal gait characteristics during transspinal stimulation at 15, 30, and 50 Hz at sub-threshold and supra-threshold stimulation intensities. We document the temporal structure of gait patterns, dynamic stability of joint movements over stride-to-stride fluctuations, and limb coordination during walking at a self-selected speed in healthy subjects. We found that transspinal stimulation (1) affects the kinematics of the hip, knee, and ankle joints, (2) promotes a more stable coordination at the left ankle, (3) affects interlimb coordination of the thighs, and (4) intralimb coordination between thigh and foot, (5) promotes greater dynamic stability of the hips, (6) increases the persistence of fluctuations in step length variability, and lastly (7) affects mechanical walking stability. These results support that transspinal stimulation is an important neuromodulatory strategy that directly affects gait symmetry and dynamic stability. The conservation of main effects at different frequencies and intensities calls for systematic investigation of stimulation protocols for clinical applications.

## Introduction

Spinal neuronal networks can generate flexible and adaptable rhythmic motor activity in absence of descending control and movement mediated afferent inputs^1–3^. These spinal neuronal networks are known as central pattern generators (CPGs) and have been extensively studied in animals under different preparations including genetic transcription factors and computational modeling^4–7^. Seminal works on the existence of mammal CPGs were the rhythmic motor discharges in a decerebrate, spinalized and deafferented cat postulated by Thomas Graham Brown (1882–1965) and his proposal of “half-centres” on each side of the spinal cord followed by Anders Lundberg (1920–2009) and Elzbieta Jankowska (1930-) works on the physiology and function of the spinal interneuronal networks and their control on stepping^8–10^. Human CPGs have been a black box for decades, giving rise to great debates regarding their existence and function^11^. Direct evidence in humans is difficult, but the similarities between the long-latency flexor reflex in L-3,4-dihydroxyphenylalanine treated spinalized animals and humans^12,13^, functional organization of interneurons mediating sensory feedback^14–16^, and spontaneous rhythmic leg movements in paralyzed persons^17,18^ support for a spinal control of stepping in humans. The deep similar modulation pattern of the soleus stretch and H-reflex in humans and mesencephalic cats during walking^19,20^, suggest that quadrupedal and bipedal locomotion share common neuronal networks regardless of the evolution of gait.

In the human isolated spinal cord, step-like phasic motor activity is generated following tonic epidural stimulation at 25–60 Hz^21^. Epidural stimulation produces either locomotor-like patterns, synchronous activation of all limb muscles, or different combinations of both^22–25^. Epidural stimulation at 5 to 15 Hz and 25 to 50 Hz generates either co-contraction or alternated activity between antagonists supporting for activation of neuronal networks for standing and walking based on frequency^23^. Similarly, transspinal (transcutaneous spinal cord) stimulation over the thoracolumbar enlargement can provide tonic excitatory inputs to spinal locomotor circuits. Transspinal stimulation at 30 Hz produces step-like movements based on muscle activation patterns in people with intact or partially injured spinal cord when legs were held in a gravity neutral position or during walking^26–28^. Transspinal stimulation with a single 1-ms pulse at supra-threshold intensities has direct actions on human spinal locomotor networks based on the profound suppression of both flexor and extensor reflexes and electromyographic (EMG) activity of knee and ankle muscles during walking^29–31^.

The function of locomotor centers during transspinal stimulation at different frequencies and intensities has never been systematically investigated before. This lack of knowledge limits our understanding on the underlying mechanisms of actions of transspinal stimulation. In this study we established the function of locomotor networks during transspinal stimulation at 15, 30, and 50 Hz at sub-threshold and supra-threshold transspinal stimulation intensities via 3D joint kinematics. Specifically, we determined (1) the temporal structure of gait patterns variability, (2) dynamic stability of joint movements over stride-to-stride fluctuations, (3) forward walking mechanical stability, and (4) intralimb-interlimb coordination computed as the spatiotemporal pattern between limb segments when transspinal stimulation was delivered at 15, 30, and 50 Hz at sub- and supra-threshold stimulation intensities during walking at a self-selected speed in healthy subjects. We hypothesized that transspinal stimulation, regardless of frequency and intensity, promotes physiological step progression and joint movements.

## Materials and methods

### Participants

Ten physically active subjects (5 female; mean age 26.7 ± 3.7 years; mean height 1.70 ± 0.2 m; and mean mass 72.7 ± 24.2 kg) with no history of neuromusculoskeletal disorder participated in the study. The experimental protocol was approved by the City University of New York (CUNY) Institutional Review Board Committee (IRB No. 2022-0003-CSI). All experimental procedures were conducted in compliance with the Declaration of Helsinki and the CUNY IRB-wide regulations and guidelines. All participants provided written informed consent before study enrolment and participation.

### Transspinal stimulation

With subjects seated, the Thoracic 10 spinous process was identified via palpation of spinal processes and anatomical landmarks. A single reusable self-adhered cathode electrode (10.2 × 5.1 cm^2^, Uni-Patch, Massachusetts, USA) was placed along the spinal processes, and covered from Thoracic 10 to Lumbar 1–2 vertebral levels. A pair of interconnected anode electrodes (same type as the cathode) was placed on either side of the iliac crests^32–34^. With subjects’ supine, hip/knee joints flexed at 30° and legs maintained in midline via external support, single 1-ms pulses were delivered by a constant current stimulator (DS7A, Digitimer, Welwyn Garden City, UK) triggered by Spike 2 scripts (1401 plus, Cambridge Electronics Design Ltd., Cambridge, UK). The position of the cathodal stimulating electrode was based on the latency, shape, and amplitude of the right and left soleus transspinal evoked potential (TEP) at increasing intensities observed on a digital oscilloscope (Tektronix, USA). After the optimal location was identified, the electrode was affixed to the skin via Tegaderm transparent film (3M Healthcare, Minnesota, USA), and maintained under pressure via a custom-made pad. For each subject while supine, the stimulation intensity that the right soleus TEP was first noted (TEP ≥ 100 mV peak-to-peak amplitude) on the oscilloscope was termed as TEP threshold^32,35^.

### Experimental data collection

**Figure 1:**
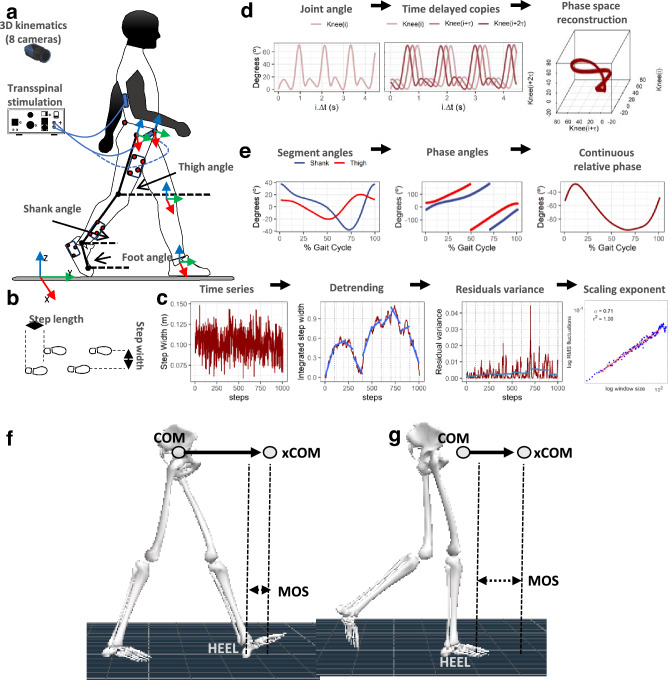
Methods for kinematic analysis during transspinal stimulation. (**a**) Experimental set up and placement of transspinal stimulation electrodes and reflective markers for tracking body segments. Subjects walked on a treadmill for 10 min while transspinal stimulation was delivered with a DS8R stimulator at 15, 30 and 50 Hz with 10 kHz carry over frequency at sub- and supra-threshold stimulation intensities. 3D kinematics were recorded for the lower limbs with an 8-camera optoelectronic system at 150 Hz. (**b**) *Step length* during walking was defined as the length between the left and right footprints at the time of heel contact. *Step width* during walking was defined as the mediolateral distance between two subsequent footprints. (**c**) Estimation of changes in step length and step width fluctuations as a function of timescale for the detrended fluctuation analysis (DFA). The demeaned and integrated spatiotemporal time series was divided into sequences of non-overlapping windows. A fitted linear regression line was subtracted from the data in each window (detrending), and the average of the local squared residuals was calculated for each window. This process was repeated for several different timescales or windows. A regression analysis was performed between the squared root of the average squared residual and the timescale to estimate the scaling exponent α-DFA. (**d**) Phase space reconstruction for the calculation of the largest Lyapunov exponent (LyE) of the joint angles using the method of delayed embedding. Optimal time delay (τ) and embedding dimension (m) parameters were calculated using the average mutual information and the false nearest neighbor algorithms. The 3D projection of the reconstructed phase space obtained with optimal embedding parameters τ = 24 and m = 3 for the right knee joint angle of a subject during control walking is shown. (**e**) Analytic signals were computed from the centered segment angles via Hilbert transformation. The continuous relative phase was calculated as the arctangent of the product of the analytic signal of the proximal angle with the conjugate analytic signal of the distal angle. The continuous relative phase estimation between the right thigh-shank coupling is shown. (**f**,**g**) Definition of the parameters used to compute margin of stability (MOS) at heel contact (**f**) and at toe-off (**g**). The margin of stability was calculated as the antero-posterior distance from the extrapolated center of mass (xCOM) to the lead heel (HEEL). Negative margin of stability means that the xCOM is ahead of the heel, while positive margin of stability means that the heel is ahead of the xCOM. COM: center of mass; xCOM: extrapolated center of mass; MOS: margin of stability.

With subjects standing upright on a treadmill, 15 retro-reflective markers of 12.5 mm diameter were placed on the anterior–superior iliac crest, sacrum, medial and lateral femoral epicondyle, medial and lateral malleolus, calcaneal tuberosity, and mid-point between the second and third metatarsals. Markers were used to define a mechanical model of pelvis, right and left thigh, shank, and foot rigid body segments (Fig. 1a). Rigid clusters of 3 or 4 retro-reflective markers were placed on the thigh, shank, and foot segments away from the anatomical landmarks to track the movement of these segments during walking^36^. The three pelvic anatomical markers were used for tracking the pelvis segment. A static calibration trial while standing on the treadmill was collected while the anatomical markers and rigid clusters were attached. Then, we removed the anatomical retro-reflective markers that were not needed, and each subject walked on a motorized treadmill (Gait Trainer, Biodex Medical Systems, Shirley, NY, USA) at their self-selected speed (3.9 ± 0.5 km/h). For each subject, 7 walking bouts of 10-min duration each were completed. Transspinal stimulation was delivered with a DS8R stimulator (Digitimer Ltd., UK). Before participants began walking on the treadmill, we checked whether transspinal stimulation at supra-threshold intensity caused discomfort or pain. Specifically, we adjusted the stimulation intensity delivered at 50 Hz to a comfortable supra-threshold level, considering its potential to induce discomfort at higher intensities compared to frequencies of 15 or 30 Hz. Participants did not report discomfort with sub-threshold intensities. The first session was walking on the motorized treadmill without transspinal stimulation. This was followed by randomized sessions during which transspinal stimulation, consisted of 1-ms pulses, was delivered at frequencies of 15, 30 and 50 Hz, with a 10 kHz intra-pulse carrier frequency, at sub-threshold (80.2 ± 27.6 mA) and supra-threshold (169 ± 47.8 mA) stimulation intensities throughout the entire session. Intensities and frequencies for transspinal stimulation were randomly assigned within and across subjects. Subjects were free to move their arms while walking on the motorized treadmill.

### 3-dimensional (3D) kinematics

The coordinates of the markers at 150 Hz sampling frequency were collected with an 8-camera optoelectronic system (Qualisys Miqus M1, Gothenburg, Sweden). Calibration residuals were on the order of < 1 mm for an active volume of approximately 4 × 2 × 2 m^3^. The 3D coordinates were reconstructed, interpolated, and labelled in Qualisys Track Manager (Qualisys, Gothenburg, Sweden). The static calibration and walking trials were then exported as C3D files and further processed using Visual3D (C-Motion Inc., Rockville, MD). The generalized cross-validation quintic splines were used for smoothing marker coordinates^37^. A mechanical model with 6 degrees-of-freedom at the hip, knee and ankle joints was created in Visual3D using the static calibration trial. The hip joint center was computed from the pelvic markers using regression equations^38^. The knee and ankle joint centers were estimated based on the kinematic analysis of marker clusters located on each joints’ distal segment during a walking trial^39^. The knee and ankle joint centers were located at the midpoint between the medial and lateral femoral epicondyle, and medial and lateral malleolus markers, respectively, both aligned with the functional rotation axis of the joints.

Segment coordinate systems were constructed according to the International Society of Biomechanics (ISB) standards, with the exception that we used the projected anatomical markers to the functional axis to define the frontal plane^40^. The ankle joint was zero degrees during the static calibration trial, referenced to the shank coordinate system. The joint movements were described with Cardan’s XYZ angular convention in accordance with the ISB standards^41^, which is equivalent to the Joint Coordinate System^42^. The medio-lateral (X) axis of the proximal segment specified the flexion and extension at the joint, the floating (Y) axis specified the adduction and abduction and was defined as the cross-product of the flexion–extension axis with the longitudinal axis, and the longitudinal (Z) axis of the distal segment specified the axial rotation. Heel contact and toe-off during walking were determined based on the kinematics data^43^.

### Ethics statement

The studies involving human participants were reviewed and approved by the Institutional Review Board of the City University of New York. All participants provided their written informed consent to participate in this study.

### Data analysis

#### Detrended fluctuation analysis

Gait spatiotemporal time series were analyzed using fractal analysis techniques to establish to what extent the temporal structure of gait patterns was affected by transspinal stimulation. Step length and step width times series (Fig. 1b) during treadmill walking were calculated as the anteroposterior or mediolateral distance between two successive footprints, measured from the heel contact of the right foot to the heel contact of the left foot. Standard deviation quantified the amount of step width and step length variability. The temporal structure in step width and step length variability in terms of persistence in fluctuations was quantified using the fractal scaling exponent (α-DFA; Fig. 1c), which was obtained from the detrended fluctuation analysis (DFA) using the average evenly spaced algorithm^44^. The minimum length of the analyzed time series (N = 884 steps) and the window size range [16, N/9] was consistent with previous recommendations for DFA calculation^45^.

The α-DFA value quantified the degree of statistical persistence (0.5 < α-DFA < 1.0) or anti-persistence (0 < α-DFA < 0.5) in the variability of step length and step width intervals. In a persistent correlation, an increase or decrease of step length or step width is more likely to be followed by another increase or decrease in the same direction, which indicates a long-range correlation. In contrast, in an anti-persistent correlation, a decrease is more likely to follow an increase and vice versa. A time series with α-DFA = 0.5 indicates that the fluctuations in the time series are uncorrelated, implying that the pattern of the time series cannot be predicted from its past values. Even if the time series is randomly permutated, it will still produce an α-DFA ≈ 0.5.

To confirm the temporal structure in the step length or step width time series, a surrogate data analysis^46^ based on permutation test was conducted to test the null hypothesis that the step width or step length fluctuations were indistinguishable from independent and identically distributed (IID) noise. Rejection of the null hypothesis would indicate that fluctuations exhibited a deterministic temporal structure. The surrogate time series were generated by random permutation of the original data and the α-DFA value was calculated from each surrogate to construct the distribution of the α-DFA value under the IID null hypothesis. The α-DFA values from the original time series and from the surrogates were compared with a two-sided rank-order test at significance level of α = 0.05. To compensate for multiple comparisons the significance level was set at α_adjusted_ = 0.005 (Bonferroni correction). For each original time series β = (2 / α_adjusted_) − 1 = 399 surrogates were generated. The p-values for the surrogate test were calculated by ranking the α-DFA values (399 surrogates + 1 original = 400 values) in numerical order and determining the rank of the DFA value of the original time series (p-value = rank / 400).

#### Largest Lyapunov exponent

The time series of the hip, knee and ankle joint flexion and extension movements were analyzed using nonlinear analysis techniques to establish to what extent the dynamic stability of joint movements over stride-to-stride fluctuations was affected by transspinal stimulation during walking (Fig. 1d). The temporal structure of the joint movement variability in terms of dynamic stability over stride-to-stride fluctuations was investigated using the largest Lyapunov exponent (LyE). The largest LyE measures the average exponential rate of divergence of the attractor’s phase trajectory (orbit) in theoretic-information terms (bits/sec). A greater rate of orbital divergence, reflected by higher positive values of the largest LyE, reflects more unstable behavior of a phase trajectory as it is repeated over time. This suggests a greater likelihood of transitioning to a more stable attractor state. A time series with largest LyE = 0 bits/sec indicates a perfectly stable, repeating pattern, which is unlikely in biological repeated movements. The reconstruction of the attractor from the joint movement time series in an m-dimensional space was based on Takens embedding theorem^47^. The appropriate delay time (τ) and embedding dimension (m) were determined for each time series using the average mutual information and global false nearest neighbor methods, respectively^48,49^. The Wolf algorithm was used to calculate the largest LyE of the respective time series^50^. In agreement with previous recommendations, the analyzed time series had a fixed number of data points across participants (N = 90,000 data points; minimum strides = 442)^51^. For the Lyapunov analysis, the number of points for time evolution was set to 3, the maximum angular orientation error between evolved and nearest neighbor points was set to 0.3 radians, the minimum distance for the selection of new nearest neighbor point was set to 0.0001, and the maximum distance for the selection of a new nearest neighbor point was set to 0.1 times the maximum length of the attractor^50,52^.

#### Continuous relative phase

The interlimb spatiotemporal pattern between the left and right thigh, and the intralimb spatiotemporal pattern between the thigh and shank, shank and foot, and thigh and foot segments were analyzed with the continuous relative phase (CRP) to establish to what extent interlimb and intralimb coordination was affected by transspinal stimulation (Fig. 1e). CRP quantifies the time shift of two segment angles S_A_ and S_B_. To model interlimb and intralimb coordination, the sagittal plane segment angles of the thigh, shank, and foot body segments were calculated relative to the right horizontal axis (Fig. 1a). Segment angles were time normalized to 101 points for the gait cycle, centered around zero, and Hilbert transformed to generate analytic signals H_A_ and H_B_^53^. The CRP between the two segment angles was calculated as the arctangent of the product of H_A_ with the conjugate of H_B_. CRP was unwrapped to resolve any discontinuities and adjusted to ± 180° for intralimb and 0º-360º for interlimb coordination. CPR from 0° or 360º to ± 180° indicates an in-phase towards an out-of-phase coordination. For the intralimb coordination, a negative CRP slope indicates that the proximal segment moves faster than the distal segment in phase space, while a positive CRP indicates that the distal segment moves faster. For the interlimb coordination, a negative CRP slope indicates that the right segment moves faster than the left segment in phase space, while a positive CRP indicates the opposite. The mean absolute relative phase (MARP) quantified the magnitude of in-phase and out-of-phase coordination. MARP was calculated by averaging the absolute CRP across gait cycles. Deviation phase (DP) quantified coordination variability. DP was calculated as the standard deviation of the CRP across gait cycles^54^. Custom-written MATLAB scripts (R2021a, MathWorks, Inc., Natick, USA) were used for data analyses.

#### Margin of stability

The extrapolated center of mass (xCOM) was used to determine the margin of stability in the anteroposterior direction during walking at heel contact and at toe off (Fig. 1f–g) to establish to what extend mechanical stability was affected by transspinal stimulation^55^. The margin of stability was defined as the anteroposterior distance between the xCOM and heel position of the leading leg at heel contact and at toe off. Changes in margin of stability during walking indicate that additional motor actions are required to preserve mechanical stability.

### Statistical analysis

The effects of 15, 30, and 50 Hz transspinal stimulation at sub- and supra-threshold intensities on joint angles, the amount (standard deviation) and temporal structure (α-DFA) of the step length and step width variability, the dynamic stability (LyE) of joint angles, and interlimb/intralimb coordination (MARP, DP) during walking were established. Linear mixed-effects models followed by a generalized linear hypothesis test for planned contrasts were used to compare transspinal stimulation groups with the control group, as well as their interaction (sub-threshold vs. supra-threshold, 15 vs. 30 Hz, 15 vs. 50 Hz, 30 vs. 50 Hz)^56,57^. Linear mixed-effects models were fitted by restricted maximum likelihood with subject as the random effect. The Westfall method was used to control the familywise type-I error rate. Cohen’s d (d < 0.20 = very small, d < 0.50 = small, d < 0.80 = moderate, otherwise interpreted as a large effect) was used to report the effect size for planned contrasts. Before model fitting, the data were preprocessed using Tukey’s boxplot method to identify and remove statistical outliers. The assumptions of the linear mixed-effects models were evaluated using graphical procedures. Variance homoskedasticity was assessed by examining plots of standardized residuals against fitted values, while residuals normality was assessed using quantile–quantile plots. The statistical analysis was conducted in R^58^. The significance level for all tests was set to α = 0.05.

## Results

### Joint kinematics

#### Effects of transspinal stimulation on ankle joint movements

**Figure 2:**
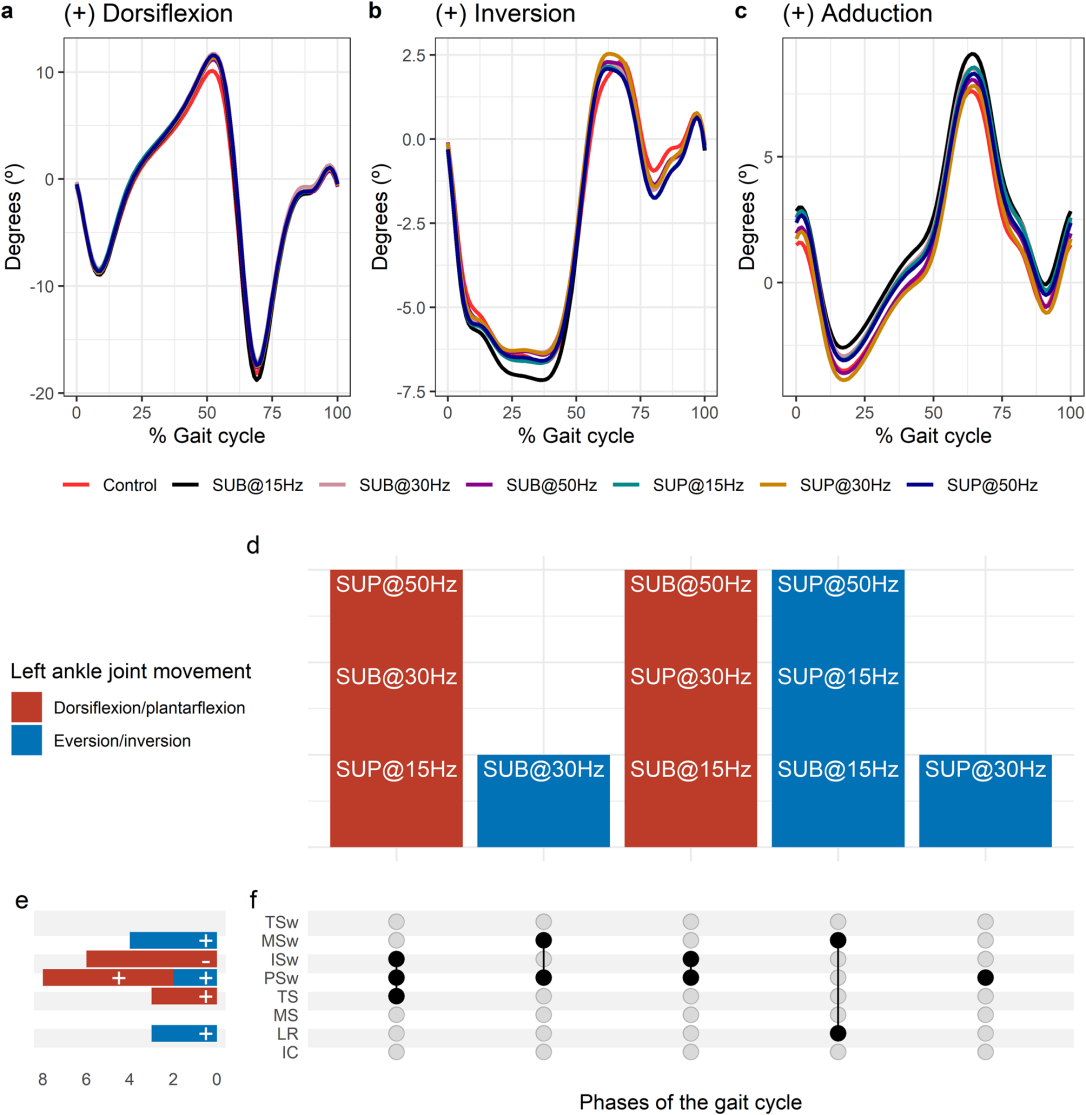
Effects of transspinal stimulation on left ankle joint movement during walking. (**a**–**c**) Left ankle joint movement with transspinal stimulation and during control walking. Solid lines represent group averages. (**d**) Vertical bars display which gait phases affected by transspinal stimulation in relation to joint movement. (**e**) Horizontal stacked bars display the cumulative number of transspinal stimulation effects on joint movement for each gait phase. Signs indicate the direction of angle change compared to control walking. (**f**) Each row corresponds to a specific gait phase, and each column represents a distinct set of gait phases. Black dots signify gait phases in which transspinal stimulation had significant effect on joint movement compared to control walking. Multiple black dots in a column indicate significant transspinal stimulation effects across different gait phases. *SUB* sub-threshold, *SUP* supra-threshold, *IC* initial contact, *LR* loading response, *MS* midstance, *TS* terminal stance, *PSw* pre-swing; *ISw* initial swing, *MSw* mid-swing, *TSw* terminal swing.

Average left ankle joint kinematics from all subjects during treadmill walking for dorsiflexion (Fig. 2a), inversion (Fig. 2b), and adduction (Fig. 2c) are shown for control conditions and during transspinal stimulation. The frequency and intensity that transspinal stimulation had significant effects on the left ankle joint movement compared to control walking are shown in Fig. 2d. Ankle eversion increased at loading response, dorsiflexion increased at terminal stance and pre-swing, inversion increased at pre-swing, and plantarflexion decreased at initial swing (Fig. 2e). The specific gait phases during which the ankle movement was significantly affected are summarized in Fig. 2f. The largest effect was observed with 30 Hz sub-threshold on dorsiflexion/plantarflexion at terminal stance (t_53_ = 3.05, p_adj_ = 0.02, d = 0.42), pre-swing (t_53_ = 5.12, p_adj_ < 0.001, d = 0.70), and initial swing (t_53_ = 3.87, p_adj_ = 0.001, d = 0.53). The largest effect on ankle eversion/inversion was observed with 30 Hz supra-threshold at preswing (t_53_ = 2.71, p_adj_ = 0.04, d = 0.37), and at loading response (t_53_ =  − 3.19, p_adj_ = 0.01, d = 0.44) and with 15 Hz supra-threshold at mid-swing (t_53_ =  − 3.77, p_adj_ < 0.001, d = 0.52).

**Figure 3:**
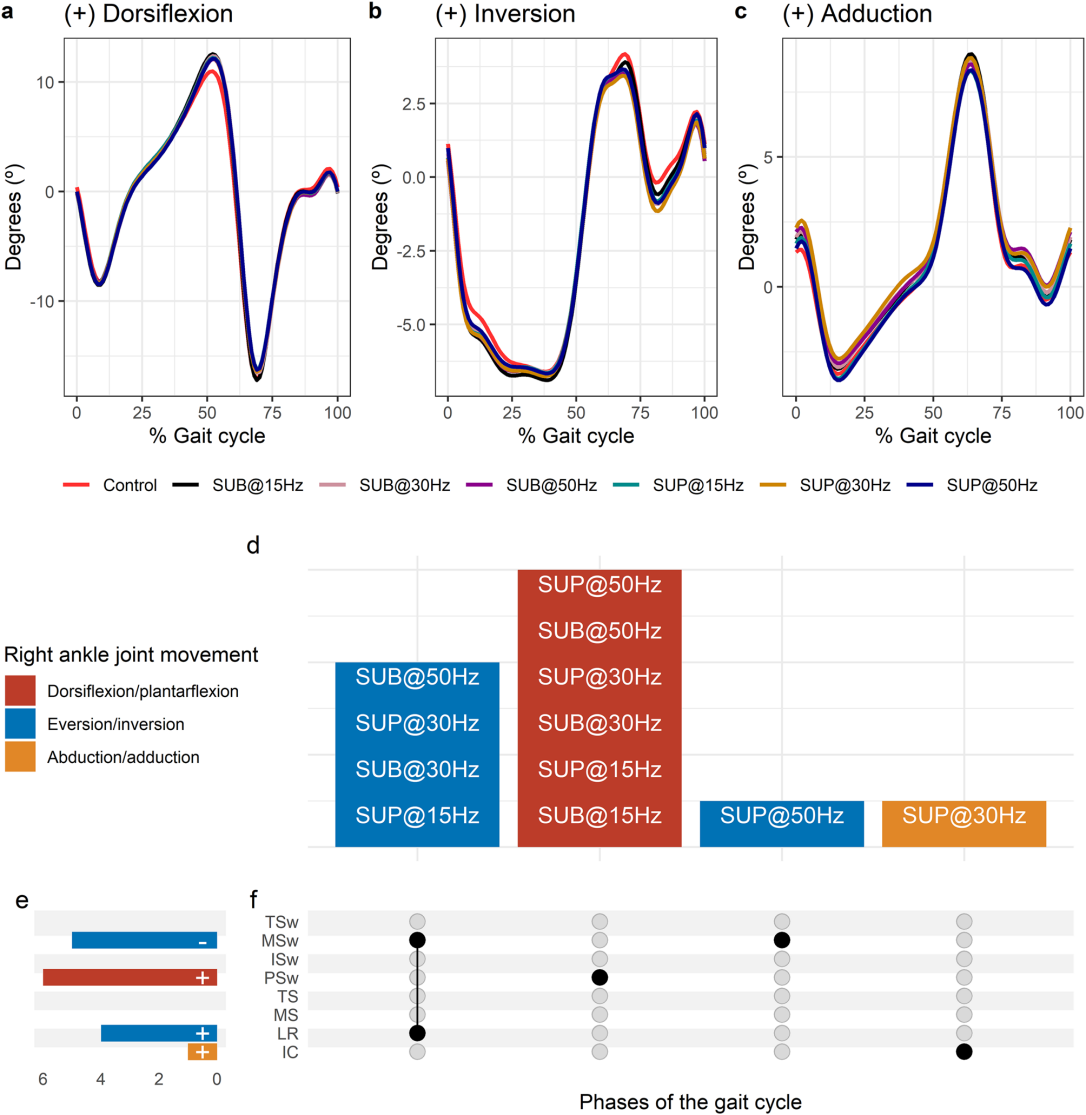
Effects of transspinal stimulation on right ankle joint movement during walking. (**a**–**c**) Right ankle joint movement with transspinal stimulation and during control walking. Solid lines represent group averages. (**d**) Vertical bars display which gait phases affected by transspinal stimulation in relation to joint movement. (**e**) Horizontal stacked bars display the cumulative number of transspinal stimulation effects on joint movement for each gait phase. Signs indicate the direction of angle change compared to control walking. (**f**) Each row corresponds to a specific gait phase, and each column represents a distinct set of gait phases. Black dots signify gait phases in which transspinal stimulation had significant effect on joint movement compared to control walking. Multiple black dots in a column indicate significant transspinal stimulation effects across different gait phases. *SUB* sub-threshold, *SUP* supra-threshold, *IC* initial contact, *LR* loading response, *MS* midstance, *TS* terminal stance, *PSw* pre-swing; *ISw* initial swing, *MSw* mid-swing, *TSw* terminal swing.

Average right ankle joint kinematics from all subjects during treadmill walking for dorsiflexion (Fig. 3a), inversion (Fig. 3b), and adduction (Fig. 3c) are shown for control conditions and during transspinal stimulation. The frequency and intensity that transspinal stimulation had significant effects on right ankle joint movement compared to control walking are shown in Fig. 3d. Ankle abduction increased at initial contact, eversion increased at loading response, dorsiflexion increased at pre-swing, and inversion decreased at mid-swing (Fig. 3e). The specific gait phases during which the ankle movement was significantly affected are summarized in Fig. 3f. The largest effect was observed with 30 Hz supra-threshold on ankle abduction/adduction at initial contact (t_53_ = 3.07, p_adj_ = 0.03, d = 0.42), eversion/inversion at loading response (t_53_ = -− 3.64, p_adj_ = 0.005, d = 0.50), and mid-swing (t_53_ = − 4.36, p_adj_ < 0.001, d = 0.60). The largest effect on ankle dorsiflexion/plantarflexion at pre-swing (t_53_ = 3.75, p_adj_ = 0.004, d = 0.52) was observed with 30 Hz sub-threshold.

#### Effects of transspinal stimulation on knee joint movements

**Figure 4:**
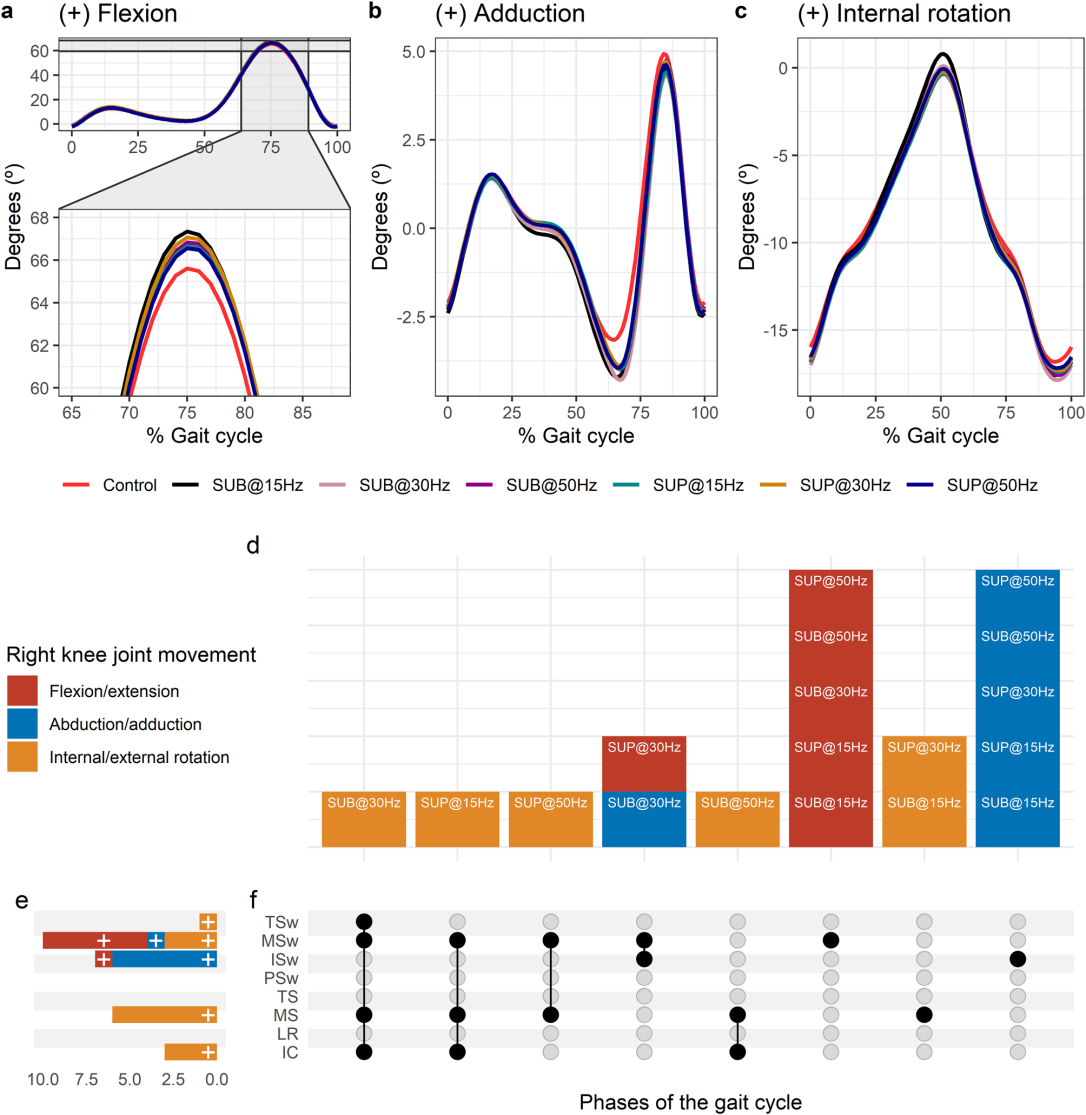
Effects of transspinal stimulation on right knee joint movement during walking. (**a**–**c**) Right knee joint movement with transspinal stimulation and during control walking. Solid lines represent group averages. (**d**) Vertical bars display which gait phases affected by transspinal stimulation in relation to joint movement. (**e**) Horizontal stacked bars display the cumulative number of transspinal stimulation effects on joint movement for each gait phase. Signs indicate the direction of angle change compared to control walking. (**f**) Each row corresponds to a specific gait phase, and each column represents a distinct set of gait phases. Black dots signify gait phases in which transspinal stimulation had significant effect on joint movement compared to control walking. Multiple black dots in a column indicate significant transspinal stimulation effects across different gait phases. *SUB* sub-threshold, *SUP* supra-threshold, *IC* initial contact, *LR* loading response, *MS* midstance, *TS* terminal stance, *PSw* pre-swing; *ISw* initial swing, *MSw* mid-swing, *TSw* terminal swing.

Average right knee joint kinematics from all subjects during treadmill walking for flexion (Fig. 4a), adduction (Fig. 4b), and internal rotation (Fig. 4c) are shown for control conditions and during transspinal stimulation. The frequency and intensity that transspinal stimulation had significant effects on right knee joint movement compared to control walking are shown in Fig. 4d. Transspinal stimulation increased external rotation, abduction, and flexion (Fig. 4e). The specific gait phases during which the right knee joint movement was significantly affected are summarized in Fig. 4f. The largest effect on internal/external rotation at initial contact (t_53_ =  − 3.64, p_adj_ = 0.006, d = 0.50), midstance (t_53_ = -3.64, p_adj_ = 0.006, d = 0.50), mid-swing (t_53_ =  − 3.30, p_adj_ = 0.02, d = 0.45), terminal swing (t_53_ =  − 3.49, p_adj_ = 0.009, d = 0.48), and on abduction/adduction at initial swing (t_53_ =  − 4.60, p_adj_ < 0.001, d = 0.63) and mid-swing (t_53_ =  − 3.32, p_adj_ = 0.01, d = 0.46) was observed with 30 Hz sub-threshold. The largest effect on flexion/extension at initial swing (t_53_ = 2.98, p_adj_ = 0.03, d = 0.41) and mid-swing (t_53_ = 4.23, p_adj_ < 0.001, d = 0.58) was observed with 30 Hz supra-threshold.

**Figure 5:**
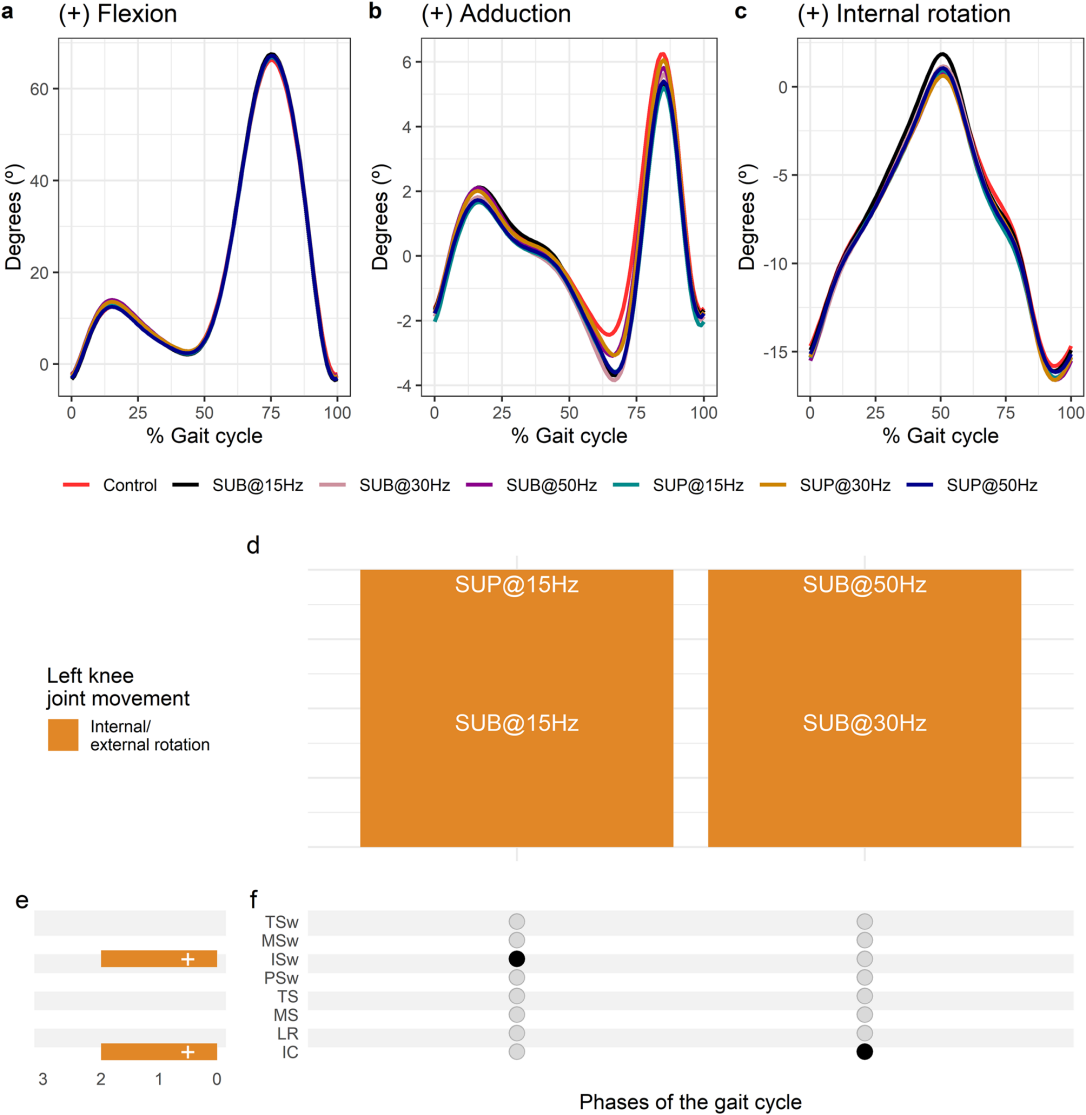
Effects of transspinal stimulation on left knee joint movement during walking. (**a**–**c**) Left knee joint movement with transspinal stimulation and during control walking. Solid lines represent group averages. (**d**) Vertical bars display which gait phases affected by transspinal stimulation in relation to joint movement. (**e**) Horizontal stacked bars display the cumulative number of transspinal stimulation effects on joint movement for each gait phase. Signs indicate the direction of angle change compared to control walking. (**f**) Each row corresponds to a specific gait phase, and each column represents a distinct set of gait phases. Black dots signify gait phases in which transspinal stimulation had significant effect on joint movement compared to control walking. Multiple black dots in a column indicate significant transspinal stimulation effects across different gait phases. *SUB* sub-threshold, *SUP* supra-threshold, *IC* initial contact, *LR* loading response, *MS* midstance, *TS* terminal stance, *PSw* pre-swing; *ISw* initial swing, *MSw* mid-swing, *TSw* terminal swing.

Average left knee joint kinematics from all subjects during treadmill walking for flexion (Fig. 5a), adduction (Fig. 5b), and internal rotation (Fig. 5c) are shown for control conditions and during transspinal stimulation. Transspinal stimulation increased external rotation at initial contact and at initial swing (Fig. 5d–f). The largest effect on internal/external rotation at initial contact was observed with 30 Hz sub-threshold (t_53_ =  − 3.01, p_adj_ = 0.03, d = 0.41), and at initial swing with 15 Hz supra-threshold (t_53_ =  − 3.70, p_adj_ = 0.005, d = 0.51).

#### Effects of transspinal stimulation on hip joint movement

**Figure 6:**
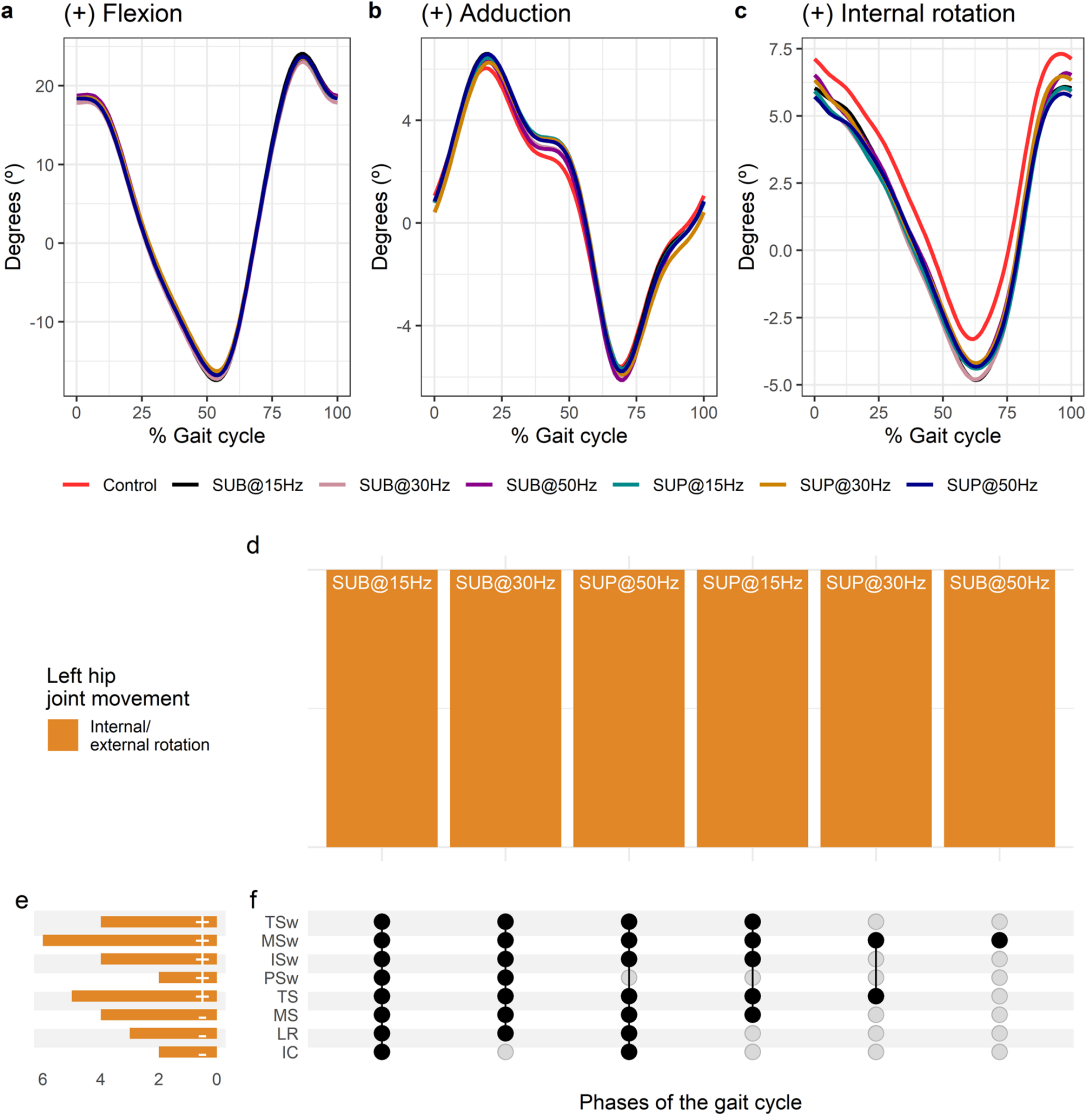
Effects of transspinal stimulation on left hip joint movement during walking. (**a**–**c**) Left hip joint movement with transspinal stimulation and during control walking. Solid lines represent group averages. (**d**) Vertical bars display which gait phases affected by transspinal stimulation in relation to joint movement. (**e**) Horizontal stacked bars display the cumulative number of transspinal stimulation effects on joint movement for each gait phase. Signs indicate the direction of angle change compared to control walking. (**f**) Each row corresponds to a specific gait phase, and each column represents a distinct set of gait phases. Black dots signify gait phases in which transspinal stimulation had significant effect on joint movement compared to control walking. Multiple black dots in a column indicate significant transspinal stimulation effects across different phases of gait. *SUB* sub-threshold, *SUP* supra-threshold, *IC* initial contact, *LR* loading response, *MS* midstance, *TS* terminal stance, *PSw* pre-swing; *ISw* initial swing, *MSw* mid-swing, *TSw* terminal swing.

Average left hip joint kinematics from all subjects during treadmill walking for flexion (Fig. 6a), adduction (Fig. 6b), and internal rotation (Fig. 6c) are shown for control conditions and during transspinal stimulation. Left hip flexion/extension and adduction/abduction were not significantly affected with transspinal stimulation. Statistically significant differences on left hip internal/external rotation between transspinal stimulation and control walking were revealed at all frequencies and intensities (Fig. 6d). Internal rotation decreased from initial contact to midstance, and external rotation increased from loading response to terminal swing (Fig. 6e,f). The specific phases of the gait cycle during which transspinal stimulation had a significant effect on left hip joint movements are shown in Fig. 6f. The largest effect was observed with 50 Hz supra-threshold at initial contact (t_53_ = − 2.88, p_adj_ < 0.05, d = 0.40), loading response (t_53_ = − 2.89, p_adj_ < 0.05, d = 0.40), and terminal swing (t_53_ = − 3.80, p_adj_ = 0.002, d = 0.52), while the largest effect with 30 Hz sub-threshold was observed at midstance (t_53_ = − 3.26, p_adj_ = 0.017, d = 0.45), terminal stance (t_53_ = − 3.83, p_adj_ = 0.003, d = 0.53), initial swing (t_53_ = − 3.51, p_adj_ = 0.008, d = 0.48), and midswing (t_53_ = − 4.18, p_adj_ < 0.001, d = 0.58) phases of gait.

**Figure 7:**
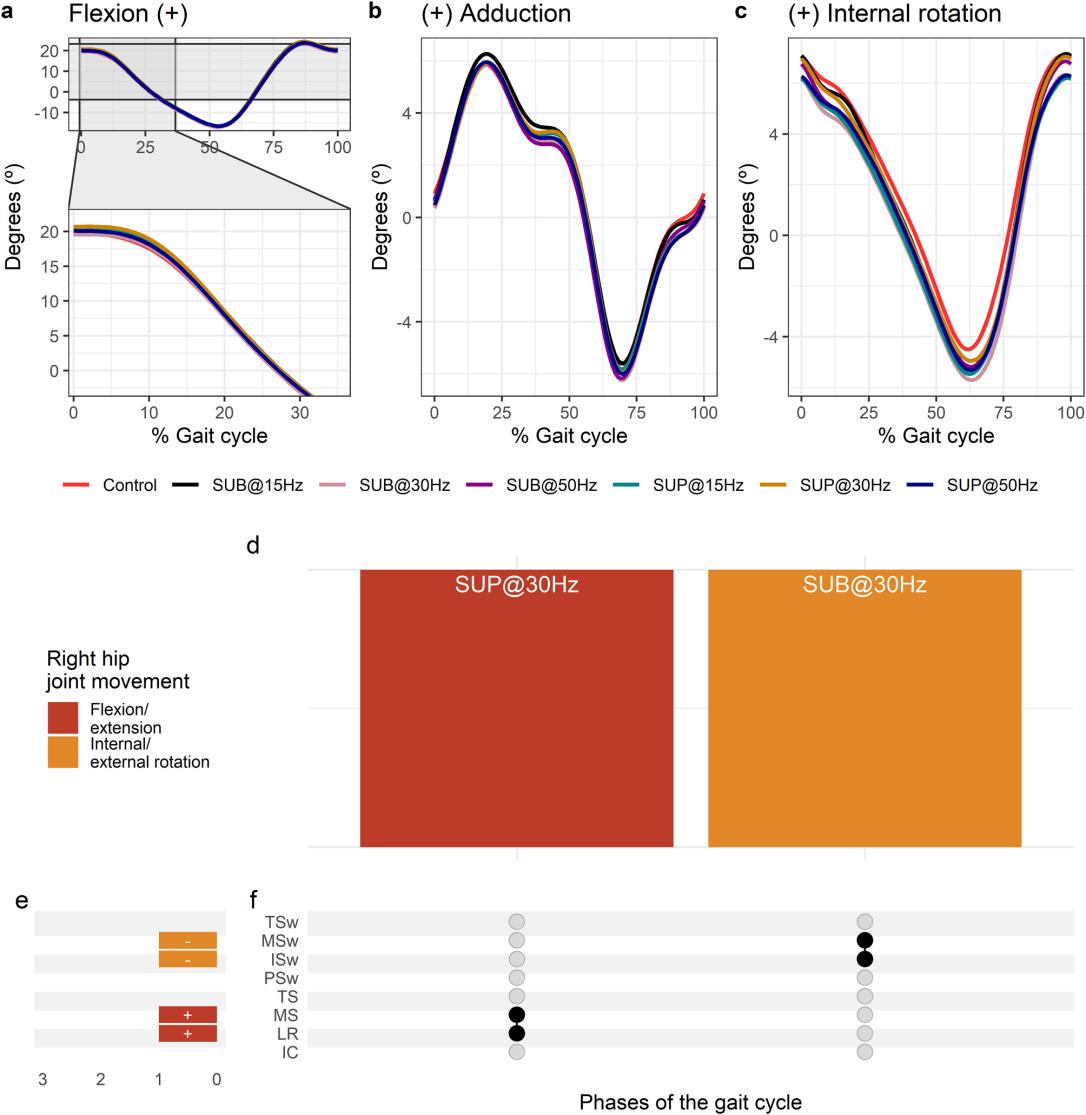
Effects of transspinal stimulation on right hip joint movement during walking. (**a**–**c**) Right hip joint movement with transspinal stimulation and during control walking. Solid lines represent group averages. (**d**) Vertical bars display which gait phases affected by transspinal stimulation in relation to joint movement. (**e**) Horizontal stacked bars display the cumulative number of transspinal stimulation effects on joint movement for each gait phase. Signs indicate the direction of angle change compared to control walking. (**f**) Each row corresponds to a specific gait phase, and each column represents a distinct set of gait phases. Black dots signify gait phases in which transspinal stimulation had significant effect on joint movement compared to control walking. Multiple black dots in a column indicate significant transspinal stimulation effects across different phases of gait. *SUB* sub-threshold, *SUP* supra-threshold, *IC* initial contact, *LR* loading response, *MS* midstance, *TS* terminal stance, *PSw* pre-swing; *ISw* initial swing, *MSw* mid-swing, *TSw* terminal swing.

Average right hip joint kinematics from all subjects during treadmill walking for flexion (Fig. 7a), adduction (Fig. 7b), and internal rotation (Fig. 7c) are shown for control conditions and during transspinal stimulation. The frequency and intensity that transspinal stimulation had significant effects on right hip joint movement compared to control walking are shown in Fig. 7d. Hip flexion increased at loading response (t_53_ = 3.49 p_adj_ = 0.009, d = 0.48) and midstance (t_53_ = 2.89, p_adj_ < 0.05, d = 0.40) with 30 Hz supra-threshold, and hip external rotation increased at initial swing (t_53_ = − 3.11, p_adj_ = 0.03, d = 0.43) and mid-swing (t_53_ = − 3.25, p_adj_ = 0.016, d = 0.45) phases of gait with 30 Hz sub-threshold (Fig. 7e,f).

### Coordination stability

Transspinal stimulation at all frequencies and intensities, except 50 Hz sub-threshold, significantly decreased left DP_Shank-Foot_ at initial swing compared to control walking (p_adj_ < 0.05). Furthermore, left DP_Shank-Foot_ decreased at mid-swing with 30 Hz and 50 Hz supra-threshold (p_adj_ < 0.05). These results suggest that transspinal stimulation promoted a more stable coordination pattern at the left ankle joint during the swing phase compared to control walking. Additionally, DP_Thigh_ decreased at pre-swing with 15 Hz sub-threshold (t_48_ = − 3.18, p_adj_ = 0.02, d = 0.46), indicating a more stable interlimb coordination during push off compared to control walking.

### Interlimb coordination

**Figure 8:**
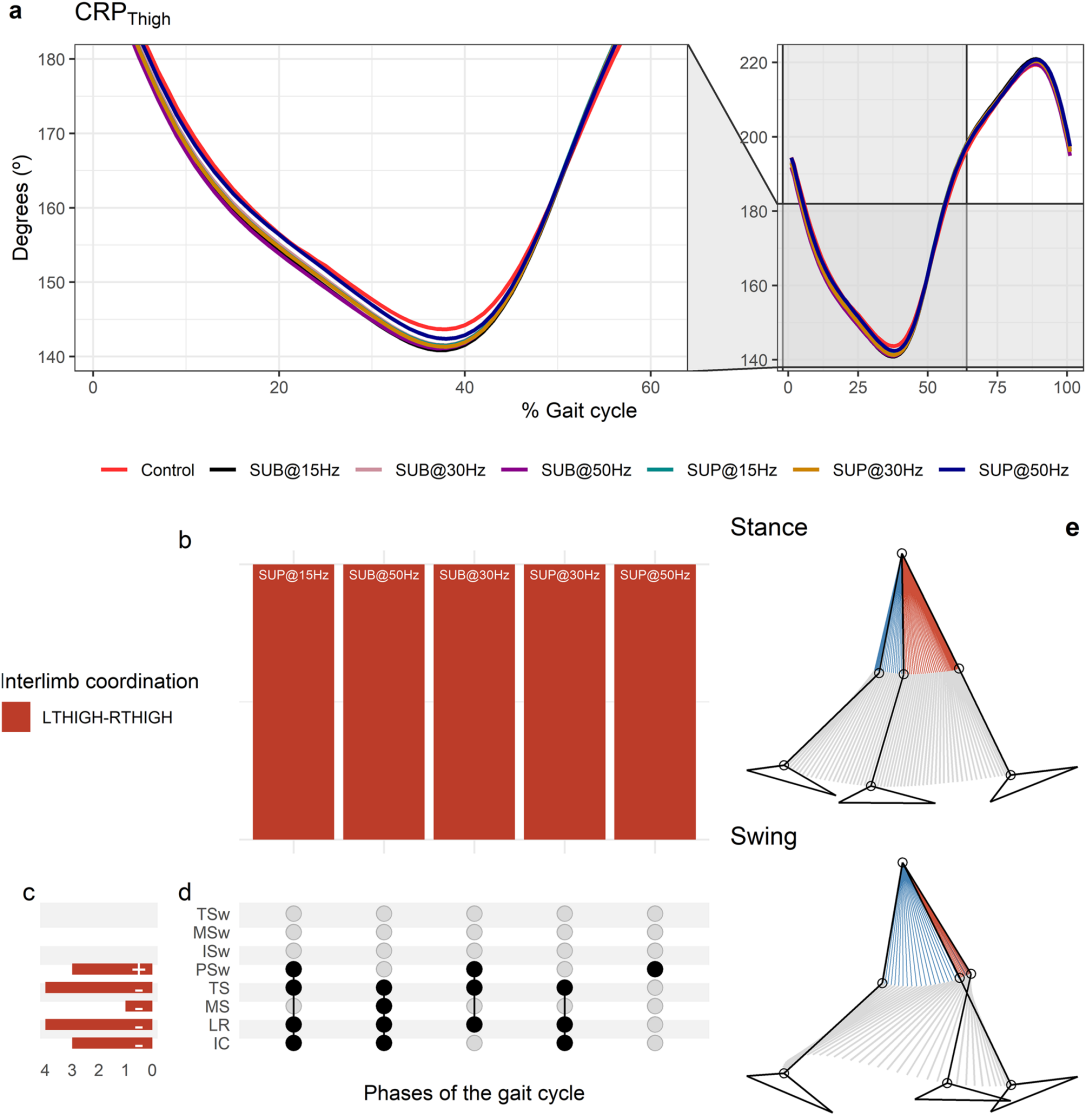
Effects of transspinal stimulation on interlimb coordination during walking. (**a**) Continuous relative phase curves for left–right thigh interlimb coordination with transspinal stimulation and during control walking. A negative slope indicates that the right segment moves faster than the left segment, while a positive slope indicates that the left segment moves faster. A value of 180° indicates an out-of-phase relationship of the coupling. Solid lines represent group averages. (**b**) Vertical bars display which gait phases affected by transspinal stimulation in relation to interlimb coordination. (**c**) Horizontal stacked bars display the cumulative number of transspinal stimulation effects on interlimb coordination for each gait phase. Both positive (+) and (−) signs represent a shift to a less out-of-phase pattern. (**d**) Each row corresponds to a specific gait phase, and each column represents a distinct set of gait phases. (**e**) The stick diagram summarizes the reversal of thighs in phase space that occurred in stance and swing phases. Black dots signify gait phases in which transspinal stimulation had significant effect on interlimb coordination compared to control walking. Multiple black dots in columns indicate significant transspinal stimulation effects across different gait phases. *SUB* subthreshold, *SUP* suprathreshold, *IC* initial contact, *LR* loading response, *MS* midstance, *TS* terminal stance, *PSw* preswing, *ISw* initial swing, *MSw* midswing, *TSw* terminal swing.

The CRP_Thigh_ was closer to 180°, indicating maintenance of the reciprocal out-of-phase coordination of the limbs during walking (Fig. 8a). At the beginning of the terminal stance that corresponds to the start of the push off phase, the left trailing thigh started to move faster than the right thigh termed as reversal. A reversal was also present at the beginning of the terminal swing which corresponds to 90% of the gait cycle. At this reversal point, the right (trailing) thigh moved faster than the left thigh. The CRP_Thigh_ curve was symmetric with respect to the double support interval, whereas legs reach an out-of-phase (180°) coordination. The relative motion of the legs produced reversals during either left or right leg push off, which corresponds to the loading response and preswing phases, respectively. The timing of the reversals in the coordination dynamics of CRP_Thigh_ (timing of maxima and minima) was similar between transspinal stimulation and control walking. Transspinal stimulation decreased CRP_Thigh_ at the first reversal (Fig. 8a).

Statistically significant MARP_Thigh_ differences between transspinal stimulation and control walking were revealed at all frequencies and intensities except with 15 Hz sub-threshold (Fig. 8b). MARP_Thigh_ decreased from initial contact to terminal swing and increased at pre-swing, indicating a less out-of-phase coordination compared to control walking—i.e. further away from 180º (Fig. 8c). The specific phases of the gait cycle whereas the significant effects of transspinal stimulation on MARP_Thigh_ were found are shown in Fig. 8d. The largest effect was observed with 50 Hz sub-threshold at initial contact (t_49_ =  − 4.64, p_adj_ < 0.001, d = 0.66), loading response (t_51_ =  − 4.82, p_adj_ < 0.001, d = 0.68), mid-swing (t_53_ =  − 3.73, p_adj_ = 0.004, d = 0.51), and terminal stance (t_52_ =  − 3.62, p_adj_ = 0.006, d = 0.50), and with 15 Hz supra-threshold at pre-swing (t_52_ = 3.76, p_adj_ = 0.004, d = 0.54) phases of gait. At initial contact and loading response, the differences on MARP_Thigh_ between stimulation intensities (sub-threshold vs. supra-threshold) were more pronounced when transspinal stimulation was delivered at 50 Hz compared to 30 Hz (t_49_ =  − 3.30, p_adj_ = 0.009, d = 0.47 and t_49_ = − 3.04, p_adj_ = 0.017, d = 0.43, respectively) or 15 Hz (t_51_ =  − 3.44, p_adj_ = 0.008, d = 0.49 and t_51_ = − 3.39, p_adj_ = 0.008, d = 0.47, respectively). The stick diagram in Fig. 8e summarizes the reversal of thighs in phase space that occurred in stance and swing phases.

### Intralimb coordination

#### Effects of transspinal stimulation on thigh-foot coordination

**Figure 9:**
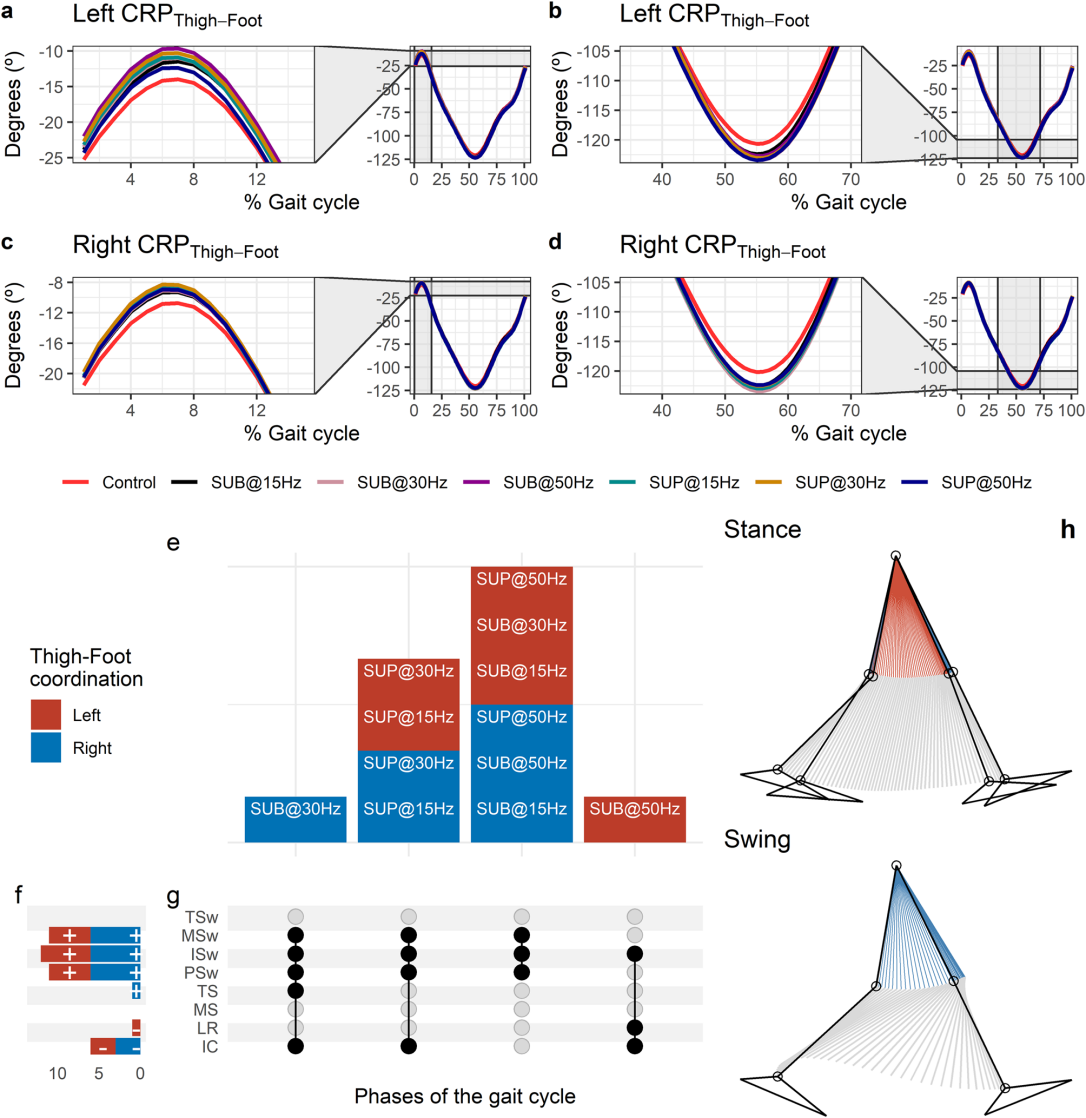
Effects of transspinal stimulation on thigh-foot coordination during walking. (**a**–**d**) Continuous relative phase curves for left and right thigh-foot intralimb coordination with transspinal stimulation and during control walking. Negative curve (°) indicates that the proximal segment is ahead of the distal segment in phase space. A negative slope indicates that the proximal segment moves faster than the distal segment, while a positive slope indicates that the distal segment moves faster than the proximal segment. A value of 0° indicates an in-phase relationship of the coupling, while a value of 180° indicates an anti-phase relationship. Solid lines represent group average. (**e**) Vertical bars display which gait phases affected by transspinal stimulation in relation to intralimb coordination. (**f**) Horizontal stacked bars display the cumulative number of transspinal stimulation effects on intralimb coordination for each gait phase. A positive (+) sign represents a shift to a greater anti-phase pattern, and a negative (−) sign a shift to a greater in-phase pattern. (**g**) Each row corresponds to a specific gait phase, and each column represents a distinct set of gait phases. Black dots signify gait phases in which transspinal stimulation had significant effect on intralimb coordination compared to control walking. Multiple black dots in columns indicate significant transspinal stimulation effects across different gait phases. (**h**) Stick figures of representative stride of a subject during walking. The red parts of the stick figures correspond to the phase when the thigh moves faster than the shank in phase space, and the blue parts when the shank moves faster than the thigh. *SUB* subthreshold, *SUP* suprathreshold, *IC* initial contact, *LR* loading response, *MS* midstance, *TS* terminal stance, *PSw* preswing, *ISw* initial swing, *MSw* midswing, *TSw* terminal swing.

The CRP_Thigh-Foot_ was closer to 0º at early stance indicating that thigh and foot exhibited a relationship closer to being in-phase (Fig. 9a,c). During initial contact the foot was moving faster than the thigh in phase space (positive slope). A reversal occurred during the loading response at about 8% of the gait cycle, where the foot achieved its maximum lead over the thigh. At this reversal point, the foot started to lose its lead, with the thigh moving faster than the foot in phase space up to pre-swing. Another reversal occurred at pre-swing at about 55% of the gait cycle, and the foot began to regain its lead over the thigh. The thigh-foot coordination became closer to being in-phase again in the late swing phase. The timing of the reversals in the coordination dynamics of CRP_Thigh-Foot_ (timing of maxima and minima) was similar between transspinal stimulation and control walking. Transspinal stimulation changed the magnitude of CRP_Thigh-Foot_ when the reversals occurred (Fig. 9b,d).

Statistically significant differences on MARP_Thigh-Foot_ between transspinal stimulation and control walking were noted at all frequencies and intensities (Fig. 9e). MARP_Thigh-Foot_ decreased at initial contact and loading response indicating a more in-phase coordination compared to control walking and increased from terminal stance to mid-swing indicating a more out-of-phase coordination (Fig. 9f). These results suggest that transspinal stimulation promotes an out-of-phase thigh-foot coordination during push off that is consistent with the hip and ankle joint kinematics we observed here ensuring adequate step progression and clearance of the foot to enter a synchronized swing phase.

The specific phases of the gait cycle that significant effects of transspinal stimulation on MARP_Thigh-Shank_ were found are shown in Fig. 9g. For the right MARP_Thigh-Foot_, the largest effect was observed with 30 Hz supra-threshold at initial contact (t_45_ =  − 3.65, p_adj_ = 0.006, d = 0.54), while the largest effect with 30 Hz sub-threshold was observed at terminal stance (t_53_ = 3.18, p_adj_ = 0.022, d = 0.46), pre-swing (t_53_ = 4.08, p_adj_ = 0.001, d = 0.58), initial swing (t_53_ = 4.47, p_adj_ < 0.001, d = 0.58), and mid-swing (t_51_ = 4.69, p_adj_ < 0.001, d = 0.66) phases of gait. For the left MARP_Thigh-Foot_, the largest effect was observed with 50 Hz sub-threshold at initial contact (t_53_ =  − 4.25, p_adj_ < 0.001, d = 0.58) and loading response (t_53_ = -3.40, p_adj_ = 0.011, d = 0.47). The largest effect for the left MARP_Thigh-Foot_ was observed with 30 Hz sub-threshold at pre-swing (t_53_ = 3.26, p_adj_ = 0.017, d = 0.45) and initial swing (t_53_ = 3.76, p_adj_ = 0.003, d = 0.52), and at mid-swing with 15 Hz supra-threshold (t_53_ = 3.61, p_adj_ = 0.006, d = 0.50). At loading response, the differences on left MARP_Thigh-Foot_ between stimulation intensities (sub-threshold vs. supra-threshold) were more pronounced when transspinal stimulation was delivered at 50 Hz compared to 30 Hz (t_52_ =  − 2.83, p_adj_ = 0.04, d = 0.40). A representative stride of a subject during walking is shown as stick diagrams in Fig. 9h. The red parts of the stick diagrams correspond to the phase when the thigh moves faster than the shank in phase space, and the blue parts when the shank moves faster than the thigh.

#### Effects of transspinal stimulation on shank-foot coordination

**Figure 10:**
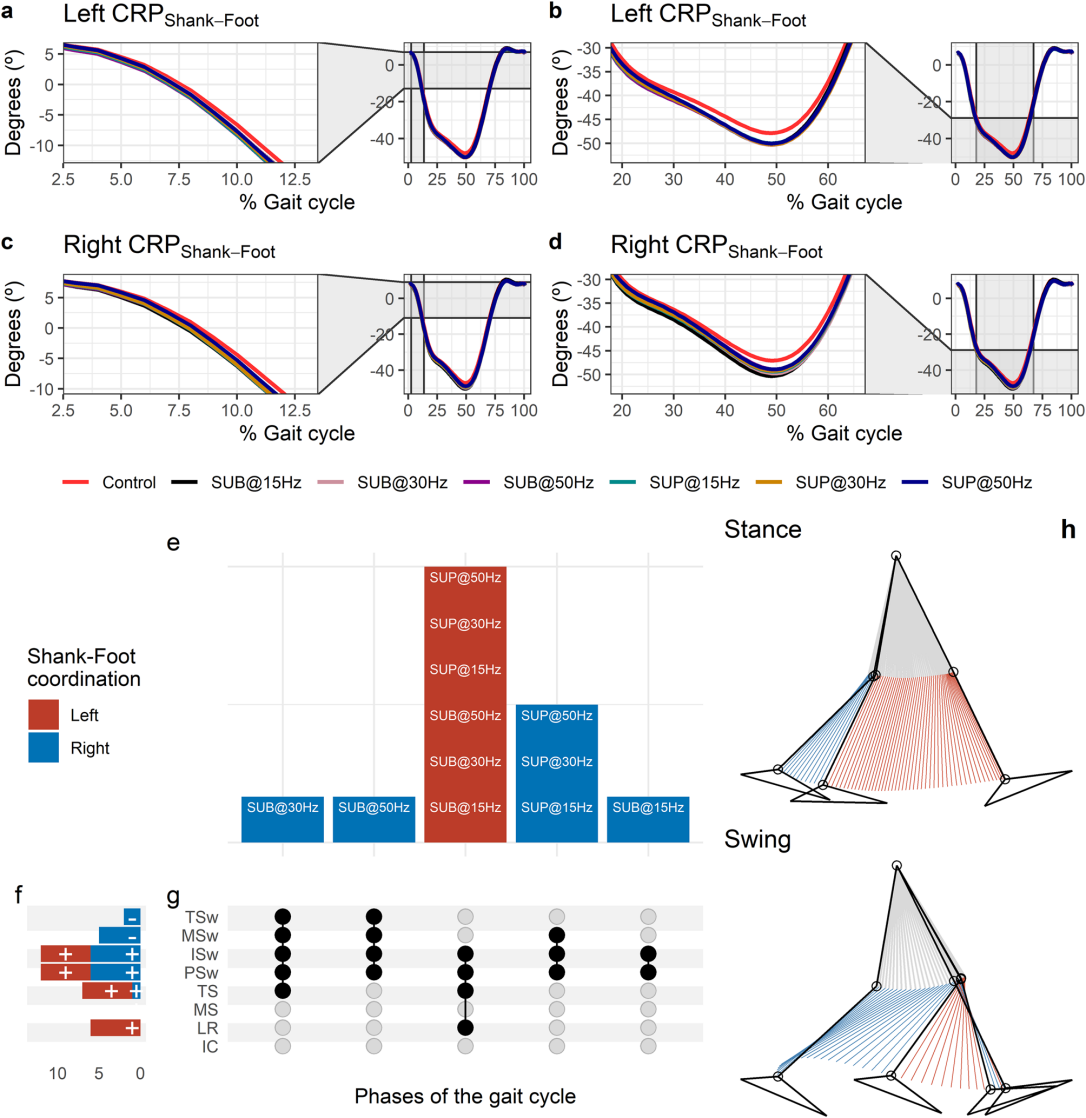
Effects of transspinal stimulation on shank-foot coordination during walking. (**a**–**d**) Continuous relative phase curves for left and right shank-foot intralimb coordination with transspinal stimulation and during control walking. Negative curve (°) indicates that the proximal segment is ahead of the distal segment in phase space. A negative slope indicates that the proximal segment moves faster than the distal segment, while a positive slope indicates that the distal segment moves faster than the proximal segment. A value of 0° indicates an in-phase relationship of the coupling, while a value of 180° indicates an anti-phase relationship. Solid lines represent group average. (**e**) Vertical bars display which gait phases affected by transspinal stimulation in relation to intralimb coordination. (**f**) Horizontal stacked bars display the cumulative number of transspinal stimulation effects on intralimb coordination for each gait phase. A positive (+) sign represents a shift to a greater anti-phase pattern, and a negative (−) sign a shift to a greater in-phase pattern. (**g**) Each row corresponds to a specific gait phase, and each column represents a distinct set of gait phases. Black dots signify gait phases in which transspinal stimulation had significant effect on intralimb coordination compared to control walking. Multiple black dots in columns indicate significant transspinal stimulation effects across different gait phases. (**h**) Stick figures of representative stride of a subject during walking. The red parts of the stick figures correspond to the phase where the thigh moves faster than the foot in phase space, and the blue parts where the foot moves faster than the thigh. *SUB* sub-threshold, *SUP* supra-threshold, *IC* initial contact, *LR* loading response, *MS* midstance, *TS* terminal stance, *PSw* pre-swing; *ISw* initial swing, *MSw* mid-swing, *TSw* terminal swing.

The CRP_Shank-Foot_ was closer to 0º indicating that the shank and foot exhibited an in-phase relationship at early stance (Fig. 10a,c). The shank was moving faster than the foot in phase space (negative slope) up to preswing, whereas a reversal occurred at about 50% of the gait cycle. At the reversal point the foot started to move faster than the shank (positive slope) until it reached a maximum at about 80% of the gait cycle. The shank-foot coordination became closer to being in-phase again at mid-swing. Transspinal stimulation increased the magnitude of the negative slope during which the shank was moving faster compared to control walking at about 12% of the gait cycle which lead to greater CRP_Shank-Foot_ when the reversal occurred compared to control walking (Fig. 10b,d). The timing of the reversal in the coordination dynamics of CRP_Shank-Foot_ (timing of minima) was similar between transspinal stimulation and control walking.

Statistically significant differences on MARP_Shank-Foot_ between transspinal stimulation and control walking were found at all frequencies and intensities (Fig. 10e). MARP_Shank-Foot_ was significantly increased at loading response and from terminal stance to initial swing indicating a more out-of-phase coordination compared to control walking and decreased at mid-swing and terminal swing indicating a more in-phase coordination (Fig. 10f). Additionally, transspinal stimulation promoted an in-phase coordination at ankle joint during mid- and terminal swing to prepare for heel contact.

The specific phases of the gait cycle whereas the significant effects of transspinal stimulation on MARP_Shank-Foot_ were found are shown in Fig. 10g. The largest effects were observed with 30 Hz supra-threshold and/or sub-threshold (p_adj_ < 0.01, 0.46 ≤ d ≤ 0.61) at loading response, terminal stance, pre-swing, and initial swing, and with 50 Hz sub-threshold at mid-swing (t_53_ = − 3.45, p_adj_ < 0.01, d = 0.48) and terminal swing (t_52_ = − 3.48, p_adj_ = 0.009, d = 0.48). This suggests that the coordination dynamics at the ankle joint during push off were affected most by transspinal stimulation delivered at 30 Hz. A representative stride of a subject during walking is shown as stick diagrams in Fig. 10h. The red part of the stick diagram corresponds to the phase where the thigh moves faster than the foot in phase space, and the blue part corresponds to the phase where the foot moves faster than the thigh.

#### Effects of transspinal stimulation on thigh-shank coordination

**Figure 11:**
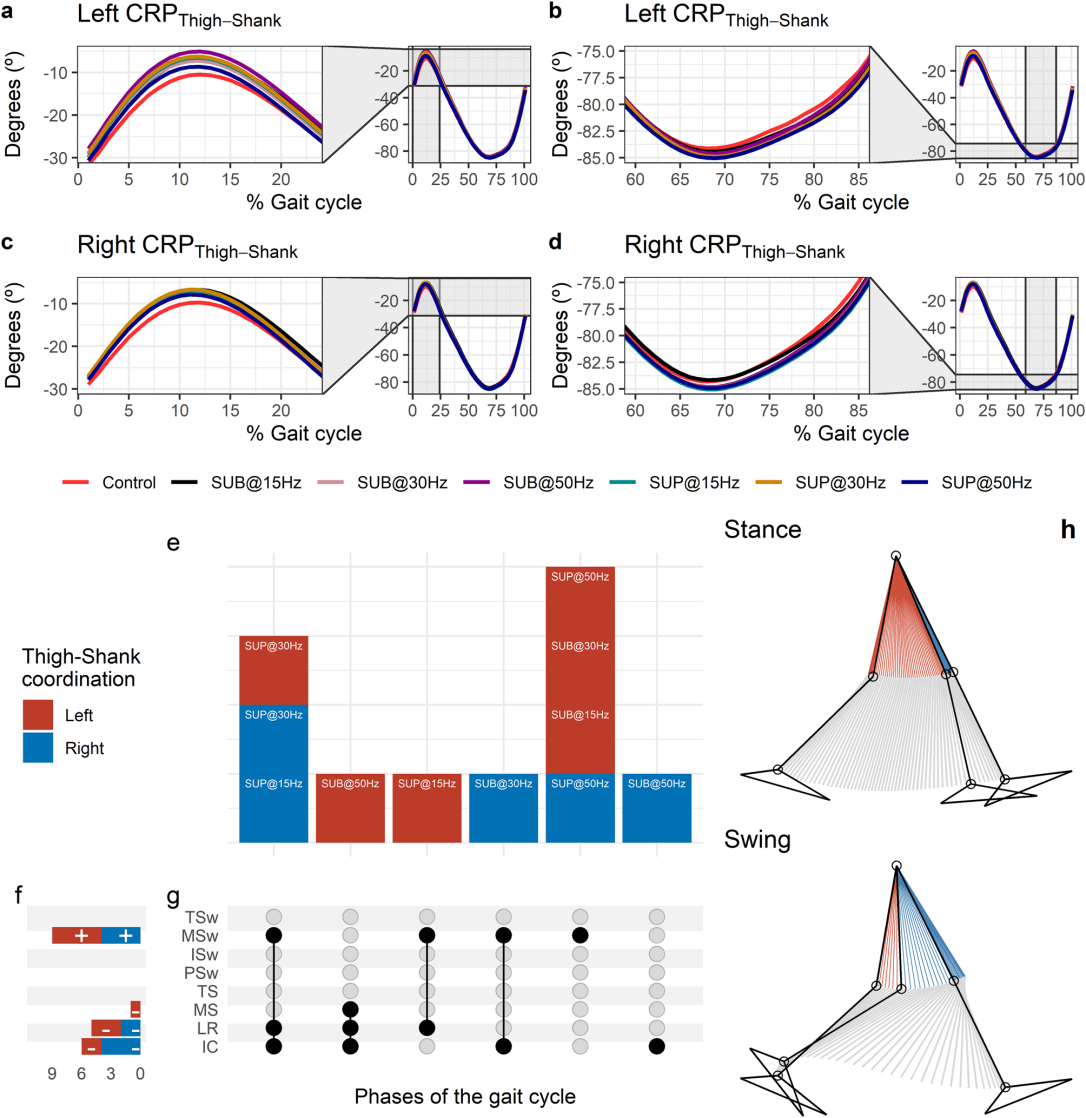
Effects of transspinal stimulation on thigh-shank coordination during walking. (**a**-**d**) Continuous relative phase curves for left and right thigh-shank intralimb coordination with transspinal stimulation and during control walking. Negative curve (°) indicates that the proximal segment is ahead of the distal segment in phase space. A negative slope indicates that the proximal segment moves faster than the distal segment, while a positive slope indicates that the distal segment moves faster than the proximal segment. A value of 0° indicates an in-phase relationship of the coupling, while a value of 180° indicates an anti-phase relationship. Solid lines represent group average. (**e**) Vertical bars display which gait phases affected by transspinal stimulation in relation to intralimb coordination. (**f**) Horizontal stacked bars display the cumulative number of transspinal stimulation effects on intralimb coordination for each gait phase. A positive (+) sign represents a shift to a greater anti-phase pattern, and a negative (−) sign a shift to a greater in-phase pattern. (**g**) Each row corresponds to a specific gait phase, and each column represents a distinct set of gait phases. Black dots signify gait phases in which transspinal stimulation had significant effect on intralimb coordination compared to control walking. Multiple black dots in columns indicate significant transspinal stimulation effects across different gait phases. (**h**) Stick figures of representative stride of a subject during walking. The red parts of the stick figures correspond to the phase where the thigh moves faster than the shank in phase space, and the blue parts where the shank moves faster than the thigh. *SUB* sub-threshold, *SUP* supra-threshold, *IC* initial contact, *LR* loading response, *MS* midstance, *TS* terminal stance, *PSw* pre-swing; *ISw* initial swing, *MSw* mid-swing, *TSw* terminal swing.

The CRP_Thigh-Shank_ was closer to 0º indicating that thigh and shank exhibited a relationship closer to being in-phase at early stance (Fig. 11a,c). A reversal occurred near the beginning of the gait cycle at about 12% of the gait cycle, where the shank achieved its maximum lead over the thigh. At this reversal point, the shank started to lose its lead, with the thigh moving faster than the shank in phase space up to initial swing. Another reversal occurred at the initial swing, at about 69% of the gait cycle, and the shank began to regain its lead over the thigh (Fig. 11b,d). The thigh-shank coordination became closer to being in-phase again in the late swing phase. The timing of the reversals in the coordination dynamics of CRP_Thigh-Shank_ (timing of maxima and minima) was similar between transspinal stimulation and control walking. Transspinal stimulation decreased CRP_Thigh-Shank_ at the first reversal (Fig. 11a,c).

Statistically significant differences on the MARP_Thigh-Shank_ between transspinal stimulation and control walking were found at all frequencies and intensities (Fig. 11e). MARP_Thigh-Shank_ decreased from initial contact to midstance (where the first reversal occurred), indicating a more in-phase coordination compared to control walking, and increased at mid-swing indicating a more out-of-phase coordination (Fig. 11f). These results suggest that transspinal stimulation promoted an out of phase coordination at knee joint during swing phase to increase foot clearance and advancement of the swinging leg. These results are consistent with the knee and ankle joint kinematics we observed in this study, which showed an increased knee flexion at mid-swing resulting from the excessive lead of shank over foot during push off and initial swing. Additionally, transspinal stimulation promoted an in-phase coordination at knee joint during initial contact and loading response, which is required to decelerate center of mass movement (power absorption) and maintain forward propulsion.

The specific phases of the gait cycle whereas the significant effects of transspinal stimulation on MARP_Thigh-Shank_ were found are shown in Fig. 11g. The largest effects were observed with 50 Hz sub-threshold on left MARP_Thigh-Shank_ at initial contact (t_53_ =  − 4.15, p_adj_ = 0.001, d = 0.60), loading response (t_53_ =  − 4.42, p_adj_ < 0.001, d = 0.61), and midstance (t_53_ =  − 2.99, p_adj_ = 0.036, d = 0.41), and with 50 Hz supra-threshold at mid-swing (t_51_ = 4.20, p_adj_ = 0.001, d = 0.59). At initial contact, the differences on left MARP_Thigh-Shank_ between stimulation intensities (sub-threshold vs. supra-threshold) were more pronounced when transspinal stimulation delivered at 50 Hz compared to 15 or 30 Hz (15 × 50 Hz: t_48_ =  − 2.69, p_adj_ = 0.043, d = 0.39; 30 × 50 Hz: t_48_ =  − 3.24, p_adj_ = 0.015, d = 0.47), loading response (30 × 50 Hz: t_52_ =  − 3.14, p_adj_ = 0.015, d = 0.44; 15 × 50 Hz: t_52_ =  − 2.83, p_adj_ = 0.03, d = 0.39). A representative stride of a subject during walking is shown as stick diagrams in Fig. 11h. The red parts of the stick figures correspond to the phase where the thigh moves faster than the shank in phase space, and the blue parts correspond to the phase where the shank moves faster than the thigh.

### Lyapunov exponent

**Figure 12:**
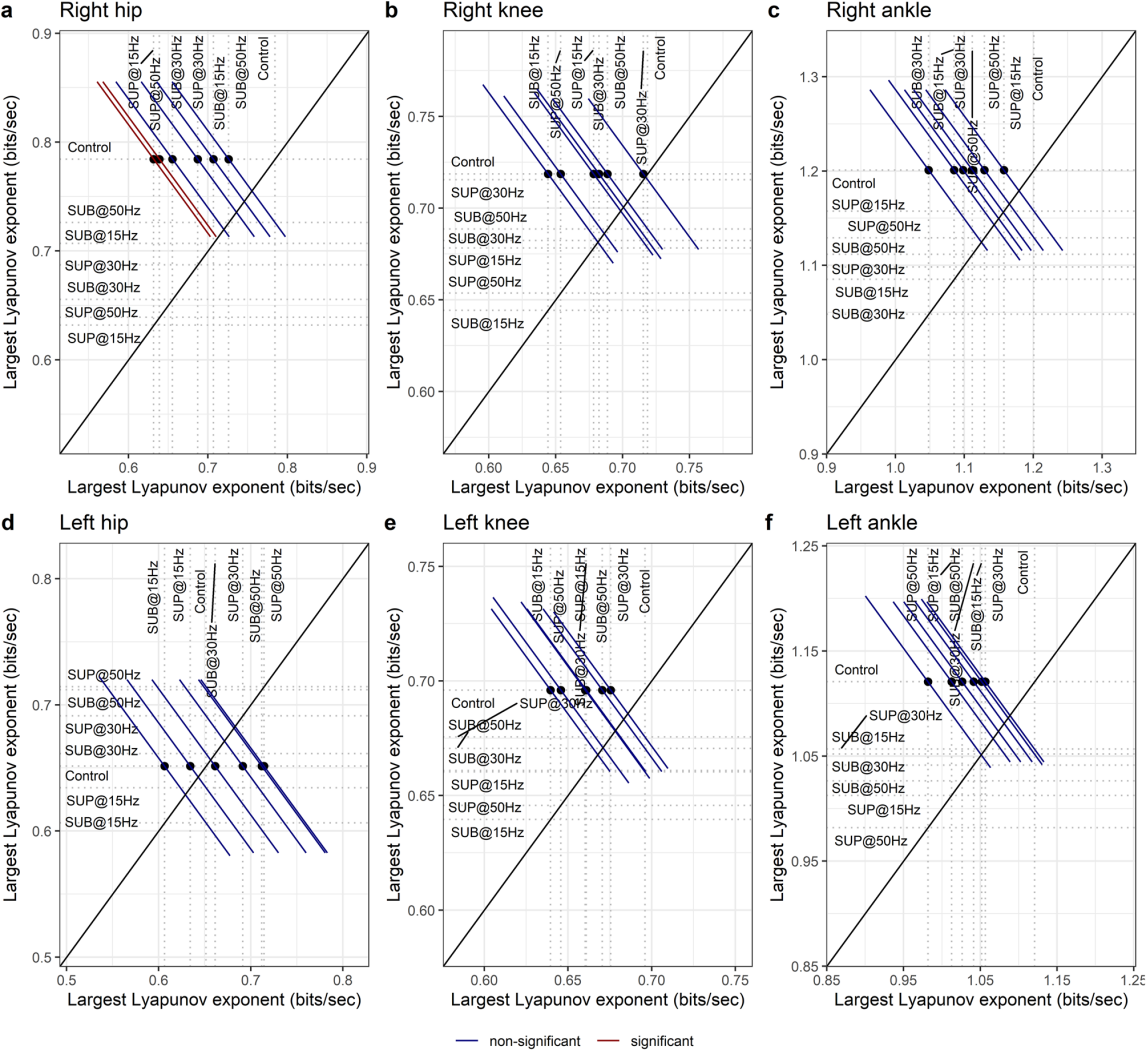
Effects of transspinal stimulation on Lyapunov exponent (LyE) of the (**a**) right hip, (**b**) right knee, (**c**) right ankle, (**d**) left hip, (**e**) left knee and, (**f**) left ankle joint. Mean-mean display of LyE values overprinted with their relative differences between transspinal stimulation and control walking. Dots show the intersection between mean values with simultaneous 95% confidence on the means. If a confidence interval crosses the diagonal line, the corresponding mean difference is non-significant. Diagonal line represents equality of means (*SUB* sub-threshold, *SUP* supra-threshold).

The LyE of the right hip flexion/extension (Fig. 12a) movement decreased with 15 Hz supra-threshold (t_54_ = − 2.94, p_adj_ = 0.039, d = 0.40) and 50 Hz supra-threshold (t_54_ = − 2.80, p_adj_ = 0.047, d = 0.38) transspinal stimulation indicating greater dynamic stability compared to control walking. Transspinal stimulation did not affect the dynamic stability of the other joint movements (Fig. 12b–f).

### Amount of gait variability

**Figure 13:**
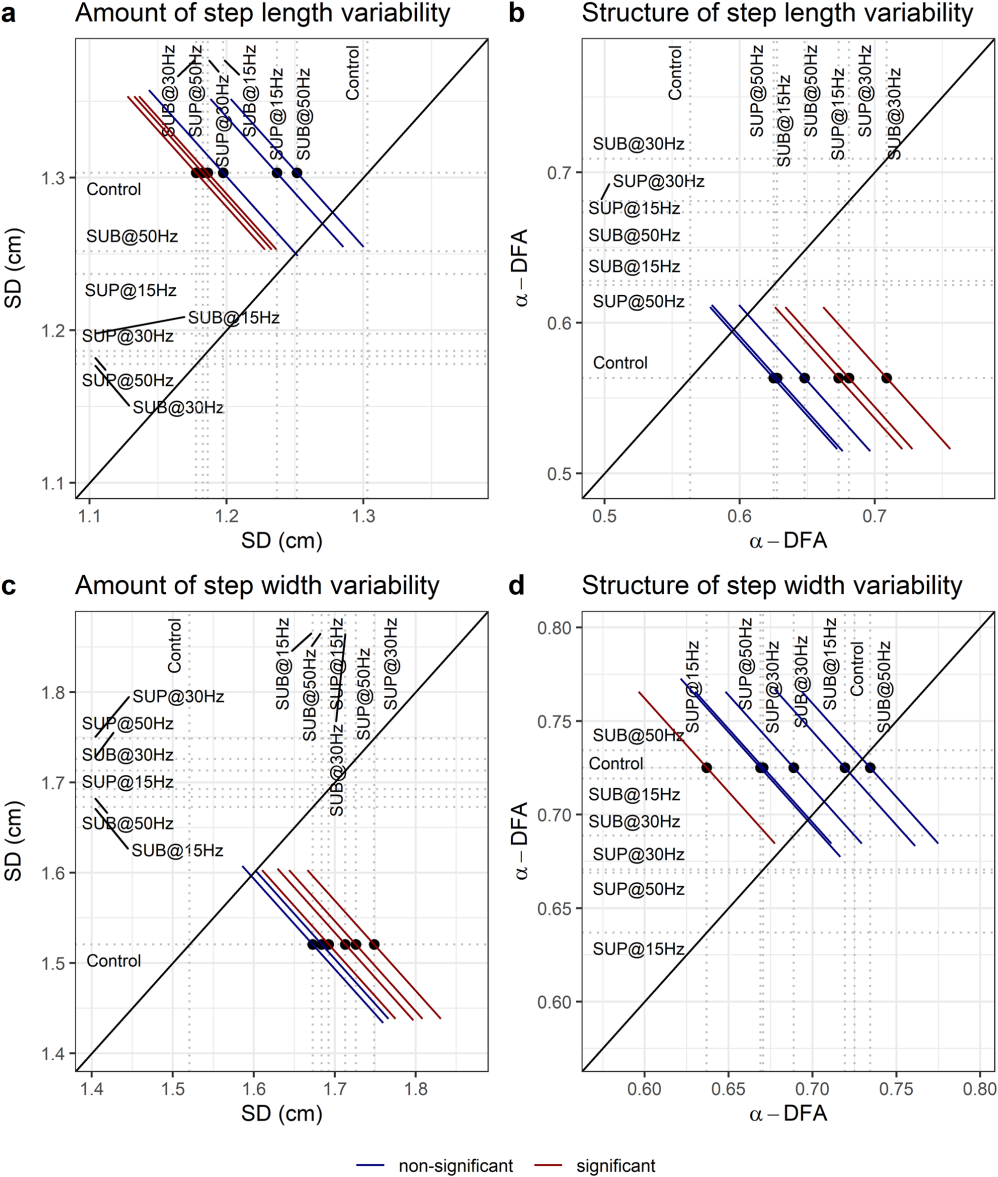
Effects of transspinal stimulation on the amount of variability of (**a**) step length and (**c**) step width fluctuations, estimated by the standard deviation (SD). Effects of transspinal stimulation on the temporal structure of variability of (**b**) step length and (**d**) step width fluctuations, estimated by the fractal exponent α-DFA. Mean-mean display of α-DFA and SD values overprinted with their relative differences between transspinal stimulation and control walking. Dots show the intersection between mean values with simultaneous 95% confidences on the means. If a confidence interval crosses the diagonal line, the corresponding mean difference is non-significant. Diagonal line represents equality of means (*SUB* sub-threshold, *SUP* supra-threshold).

The amount of step length variability decreased with 30 Hz sub-threshold (t_48_ = − 3.43, p_adj_ = 0.011, d = 0.49), 30 Hz supra-threshold (t_48_ = − 3.19, p_adj_ = 0.014, d = 0.46), and 50 Hz supra-threshold (t_48_ = − 3.29, p_adj_ = 0.014, d = 0.47) compared to control walking (Fig. 13a). The amount of step width variability increased at all frequencies and intensities (p_adj_ < 0.04, 0.38 ≤ d ≤ 0.54), except with 15 Hz sub-threshold (Fig. 13b).

### Detrended fluctuation analysis

The temporal structure of step length fluctuations became more persistent with 30 Hz sub-threshold (t_51_ = 4.24, p_adj_ < 0.001, d = 0.59), 30 Hz supra-threshold (t_51_ = 3.42, p_adj_ = 0.007, d = 0.48), and 15 Hz supra-threshold (t_51_ = 3.20, p_adj_ = 0.017, d = 0.45) compared to control walking (Fig. 13c). The temporal structure of step width fluctuations became less persistent with 15 Hz supra-threshold (t_49_ = − 2.98, p_adj_ = 0.03, d = 0.43) compared to control walking (Fig. 13d). The difference on the temporal structure of step width variability between stimulation intensities (sub-threshold vs. supra-threshold) was significant (t_49_ = 3.03, p_adj_ = 0.033, d = 0.43).

#### Surrogation analysis

**Figure 14:**
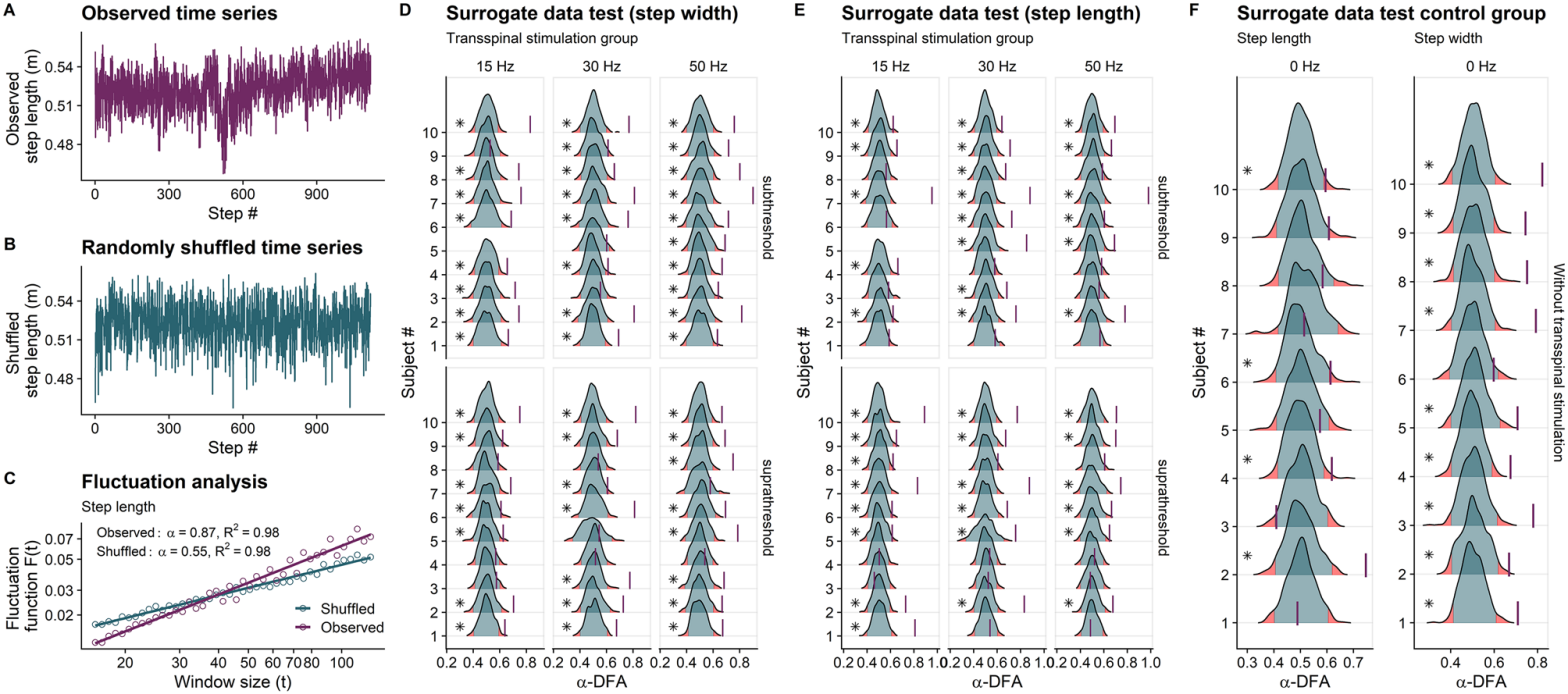
Surrogation analysis. Surrogate data for step width and step length time series for each subject (#1–10) using the fractal scaling exponent (α-DFA) obtained from detrended fluctuation analysis (DFA) as the discriminating statistic. Ten-minute step length time series before (**a**) and after random permutation (**b**), and detrended fluctuation analyses (**c**) are shown for a representative walking trial with supra-threshold transspinal stimulation delivered at 15 Hz. The solid-colored lines in (**c**) are the best fit used to calculate the α-DFA value of the original and permuted time series. For the surrogate data test, the step width and step length time series of each walking trial was randomly permutated 399 times and the α-DFA value was calculated each time. The distribution of the surrogate α-DFA values (399 data) for each walking trial is shown in (**d**) for the step width and in (**e**) for the step length time series. (**f**) Step length and step width without transspinal stimulation for each subject. The α-DFA value of the original time series is shown with a vertical line, along with the probability density functions and the quantiles (2.5% and 97.5%) of the surrogates. Asterisks indicate that the α-DFA value for the original time series was significantly greater than α-DFA values for the corresponding surrogate time series, indicating a significant difference from uncorrelated white noise (*p ≤ 0.005).

A representative step length time series is shown in Fig. 14a, while a random permutation surrogate obtained by randomly shuffled the time series is shown in Fig. 14b. The DFA analysis of step length time series and its surrogate are depicted in Fig. 14c. The DFA analysis of the original step length time series revealed persistent fluctuations around its mean value, indicating the presence of long-range correlations (α-DFA = 0.87). On the other hand, when the step length time series is randomly permutated, the resulting surrogate shows α-DFA values close to 0.5 (specifically α-DFA = 0.55). This indicates that the random permutation has disrupted the temporal long-range correlations observed in the original step length time series, resulting in uncorrelated noise. The distributions (i.e., null) of the 399 α-DFA values for the step width and step length surrogates for each transspinal stimulation group and subject are shown in Fig. 14d and e, respectively. The corresponding step length and step width without stimulation are indicated in Fig. 14f for each subject. For most subjects, the percentile of the α-DFA value of the original time series, denoted by a vertical line, was equal to or larger than 97.5%, indicating a significant difference from uncorrelated white noise (p ≤ 0.025). This confirmed that most subjects exhibited deterministic characteristics of persistent fluctuations in both step width and step length time series during walking with sub-threshold or supra-threshold transspinal stimulation delivered at the different frequencies.

### Margin of stability

**Figure 15:**
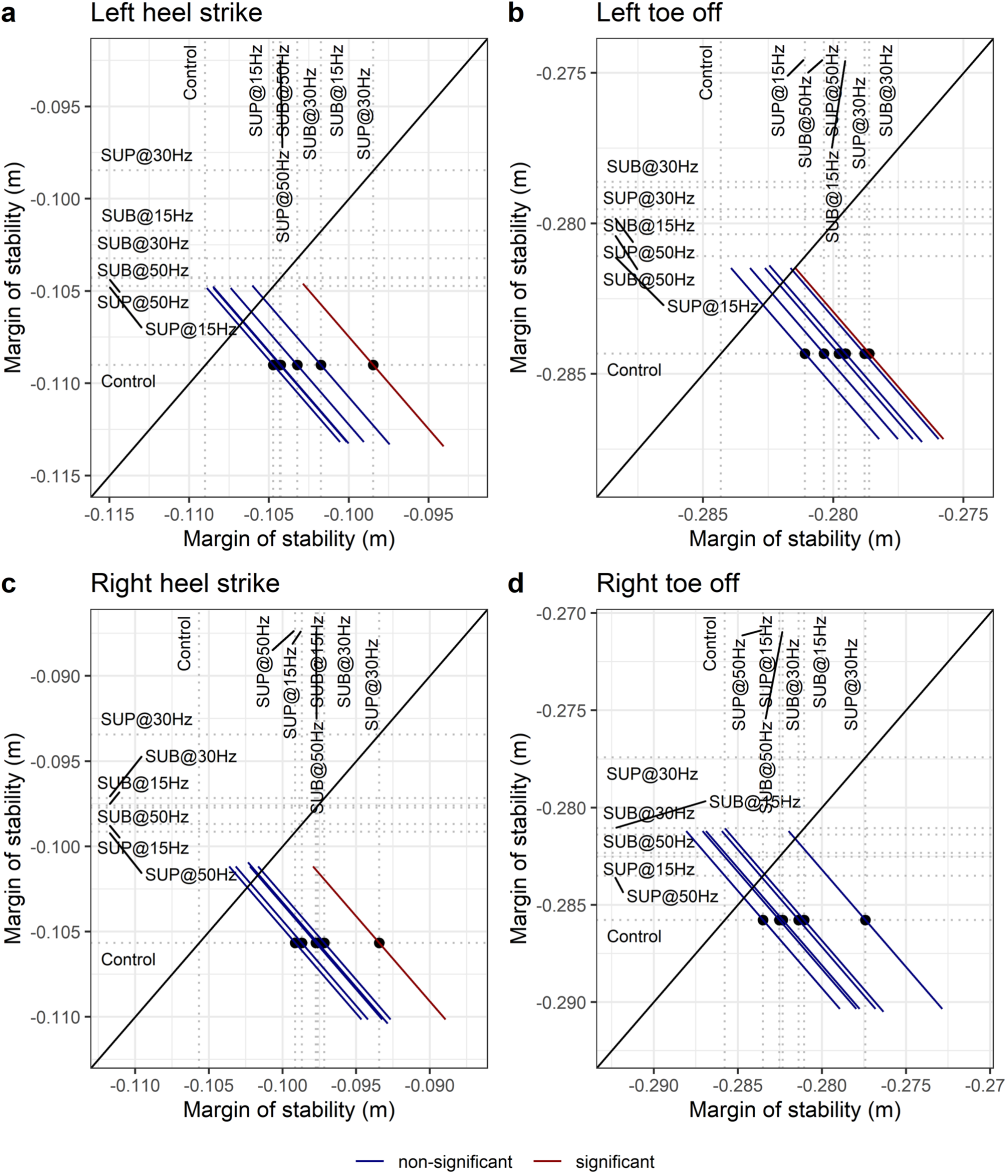
Effects of transspinal stimulation on margin of stability at (**a**) left heel contact, (**b**) left toe off, (**c**) right heel contact, and (**d**) right toe off. Mean-mean display of margin of stability values overprinted with their relative differences between transspinal stimulation and control walking. Dots show the intersection between mean values with simultaneous 95% confidences on the means. If a confidence interval crosses the diagonal line, the corresponding mean difference is non-significant. Diagonal line represents equality of means. *SUB* sub-threshold, *SUP* supra-threshold.

The margin of stability decreased at the heel strike for the left (t_49_ = 3.28, p_adj_ = 0.016, d = 0.47) and right (t_50_ = 3.73, p_adj_ = 0.004, d = 0.53) legs with 30 Hz supra-threshold stimulation compared to control walking (Fig. 15a,c), while no significant effects were observed during the toe off of the right and left legs (Fig. 15b,d). These results suggest that transspinal stimulation promoted the activity required to continue walking, which is consistent with the increased out-of-phase found at thigh-foot coordination during push off (Fig. 9b,d), the increased out-of-phase at ankle joint during push off by slowing down the foot rotation (Fig. 10b,d), and the increased dorsiflexion at terminal stance and decreased plantarflexion at preswing found at ankle joint kinematics results (Figs. 3, 4). The results suggest that mechanical stability was affected most by transspinal stimulation delivered at 30 Hz.

## Discussion

This is the first systematic study that provides evidence on how transspinal stimulation at different frequencies and intensities affects the human locomotor rhythm. Transspinal stimulation over the thoracolumbar region at sub-threshold and supra-threshold paresthesia intensities with 15, 30, and 50 Hz frequencies (1) affected the kinematics of the hip, knee, and ankle joints, (2) promoted a more stable coordination at the left ankle, (3) affected interlimb coordination of the thighs, and (4) intralimb coordination between thigh and foot, (5) promoted greater dynamic stability of the hips, (6) increased the persistence of fluctuations in step length variability, and lastly (7) affected the mechanical stability of walking. Our novel findings support that transspinal stimulation is an important neuromodulatory strategy that directly affects gait symmetry and dynamic stability.

Transspinal stimulation at 30 Hz suprathreshold decreased the margin of stability during walking (Fig. 15a–c). The margin of stability is a measure that reflects the relationship between the center of mass position and velocity relative to foot placement and is directly related to mechanical stability of gait^55,59^. The margin of stability predicts the point on the ground that the foot will be placed before the body comes to a standstill. While walking at steady speed, a forward-stable gait without excessive spatiotemporal fluctuations is achieved by placing the foot behind that predicted point ensuring negative margin of stability, for example, through constant offset control^55^. Less negative margin of stability during walking with transspinal stimulation indicates reduction in mechanical stability (less resistance to center of mass redirection) and facilitation of neural control because less volitional mechanical impulse would be required to redirect the center of mass velocity compared to control walking^60^. Redirection of center of mass velocity during steady speed walking requires negative work at the ankle and hip to slow down the center of mass at terminal stance followed by positive work at the ankle at pre-swing^61,62^.

Ankle musculature generates minimal power at terminal stance, whereas negative mechanical work is primarily performed, and elastic strain energy is stored in the tendinous tissues^63,64^. A possible way to amplify ankle power at push off is through direct effects on the functional operation of the CPG by increasing triceps surae muscle activity through locomotor Ib facilitatory pathways exerted from medial gastrocnemius onto soleus motoneurons^65–67^. During walking, whereas a stretch-recoil cycle is performed by the tendinous tissues in ankle extensors^68^, prolonged dorsiflexion and thus tibial progression before push off can further increase the elastic recoil and thus amplify power output^69^. Indeed, increased out-of-phase thigh-foot coordination during push off (Fig. 9b,d) coincided concomitantly with increased out-of-phase at ankle joint (Fig. 10b,d), slowing down the foot rotation, and increased dorsiflexion at terminal stance (Figs. 3, 4). Specifically, in the left ankle transspinal stimulation mostly with 30 Hz sub-threshold and supra-threshold facilitated walking by increasing the ankle eversion at loading response, dorsiflexion at terminal stance and pre-swing, and decreasing plantarflexion at initial swing (Fig. 2e). Accordingly, it seems that transspinal stimulation promotes ankle control strategies linked to the extensor CPG.

Push off may also be facilitated by changes in the hip joint. The biarticular nature of muscles spanning the hip and knee joints, and the monosynaptic heteronymous connections between thigh flexor afferents and ankle extensor muscles^70^, support the physiological mechanism underlying the kinematics changes we observed here. The right hip flexion increased at loading response and mid-stance while hip external rotation increased at initial and mid-swing with 30 Hz regardless of intensity. Moreover, the increased knee flexion at early and mid-swing phases with 30 Hz suprathreshold supports further the progression of the step cycle, and that transspinal stimulation may have access to both the flexor and extensor CPGs.

Interlimb (right-left thighs) and intralimb (shank-foot, thigh-foot) coordination provide significant information on the function of the locomotor rhythm. Coordination between joints of the same leg, and between joints of both legs is controlled by commissural interneurons with their axons projecting to the contralateral side of the spinal cord^71^, and propriospinal neurons that modulate sensory information and carry temporal information for gait control^72–74^. These spinal neuronal circuits organize the limb movements and thereby transform rotations in joint into the desired walking patterns. Transspinal stimulation at 30 Hz supra-threshold promoted an out-of-phase thigh-foot coordination from terminal stance to mid-swing including push-off, and out-of-phase shank-foot coordination at loading response and from terminal stance to initial swing compared to control walking (Fig. 10f). Transspinal stimulation decreased the mean absolute relative phase of the thigh from initial contact to terminal swing and increased at pre-swing, indicating a less out-of-phase coordination compared to control walking. These effects were apparent with 50 Hz sub-threshold and 15 Hz supra-threshold, supporting for variable effects on intralimb coordination based on the intensity and frequency of transspinal stimulation. Transspinal stimulation increased out-of-phase coordination at the ankle joint during push off by slowing down the foot rotation, which is consistent with an increased dorsiflexion at terminal stance and decreased plantarflexion at pre-swing found at ankle joint kinematics results (Figs. 3, 4). These results further support that transspinal stimulation affects locomotor gait patterns by affecting interlimb and intralimb coordination and thus may be useful in neurological disorders that limb coordination is impaired.

Important spatiotemporal gait parameters are that of step length and step width variability, which reflect the overall physiological function of the nervous system and become pathological in neurological disorders. 30 Hz sub-threshold and 30/50 Hz supra-threshold transspinal stimulation decreased step length variability and increased that of step width (Fig. 13). These changes suggest that transspinal stimulation contributes to greater passive stability in the forward direction, accompanied by an increased reliance on supraspinal control for lateral stability. In silico simulations, physical biped-legged models, and experiments involving human walking, revealed that foot placement in forward direction is dependent mostly on the biomechanical properties of the body and their mechanical interaction with the environment^59,75–79^. On the other hand, lateral foot placement relies on an active supraspinal/spinal sensory feedback control^59,75–79^. Greater step width variability indicates increased effort of active control required for lateral stabilization^75,78,80,81^. The decrease in step length variability indicates greater contribution of passive stabilization of forward dynamics involving spinal control and simple reflex actions. The decrease on Lyapunov exponents on right hip joint corroborates the greater forward stability with 50 Hz supra-threshold stimulation. The increase in step width variability with transspinal stimulation could indicate an altered state of integration of common synaptic drive, partly from the state of the center of mass^82–84^, which is pertinent to both push off and foot placement feedback control. Based on this theoretical framework, a possible push off modulation did not decrease the need for active control to stabilize lateral motion through foot placement.

The push off mechanism works synergistically with foot placement to stabilize walking in both forward and lateral directions^61,80,81,85^. Lateral stability is partly modulated by changes in the ankle movement of the trailing leg observed during push off, for example with an increase in dorsiflexion at mid-stance observed with transspinal stimulation. However, push off modulation of lateral stability is coupled to forward stability and to step length fluctuations^85^. While walking without transspinal stimulation, the fractal scaling exponent of step length variability decreased (Fig. 13), reflecting nearly uncorrelated fluctuations^86,87^. By contrast, transspinal stimulation induced long-range correlations, suggesting that subjects adopted a control mechanism aimed at regulating step length similarly to what was observed for step width, which involves the persistence of an increase or decrease in step fluctuations across multiple consecutive steps before reversing. The observed temporal correlation suggests that step length and step width fluctuations were not controlled based only on step-by-step corrections, and probably reflects an increase on the modulation coupling between step width and step length with transspinal stimulation. The surrogation analysis revealed significant differences in step width and step length variability between the original data and their surrogate counterparts. This suggests deterministic properties in the fluctuations of both step width and step length with transspinal stimulation. Transspinal stimulation increased the deterministic behavior for step length fluctuations. The deterministic properties of the gait variability with transspinal stimulation are important because it promotes gait stability by allowing a successful adaptation of foot placement to the small inherent perturbations during walking.

Transspinal stimulation has been used at different frequencies and intensities to improve standing and walking abilities in neurological disorders, but systematic investigations to define the optimal frequency and intensity per targeted outcome are lacking. For example, transspinal stimulation at frequencies varying from 0.2 to 30 Hz promoted self-assisted standing and upright trunk posture with minimal or no external assistance^88,89^. Transspinal stimulation at 30 Hz changed the left hip-knee coordination in 2 out of 3 subjects with spinal cord injury, increased the hip range of movement by 8° in another subject^27^, and spatiotemporal gait characteristics in a motor incomplete person^90^. Frequencies from 5 to 15 Hz have been proposed to be optimal for recovery of standing because they produce bilateral leg extension, while frequencies from 25 to 50 Hz have been proposed to be optimal for recovery of walking because they evoke rhythmic alternated flexion–extension movement of the legs following epidural stimulation (reviewed in Ref.^91^). However, these conclusions are based on data from a few subjects and a comprehensive investigation is thus warranted. Correspondingly, in this study we found significant effects with all frequencies and intensities. More importantly, joint movements and joint coordination changed in all gait phases with transspinal stimulation (supplementary Fig. S1). This finding is consistent with our recent report on increased locomotor EMG activity of biarticular knee muscles during walking with transspinal stimulation^92^. A systematic approach involving animal and human research as well as simulation models is needed to design stimulation protocols that can benefit gait asymmetry and impaired dynamic balance in upper motoneuron lesions.

## Future perspectives and limitations

The detailed kinematic analysis of leg movement during treadmill walking suggested that transspinal stimulation at frequencies ranging from 15 to 50 Hz and intensities from sub-threshold to supra-threshold affected spatiotemporal parameters of gait, leg coordination, and dynamic stability. The well-established phase-dependent amplitude modulation of locomotor EMG activity^93,94^ and adaptation of lumbosacral motoneuron activation^29,30^ suggests the need for concomitant analysis of locomotor EMG activity along with kinematic and kinetic analysis of standing and walking with different transspinal stimulation protocols. Our next step is to delineate the muscle synergies during walking, and the ground reaction forces exerted during standing following transspinal stimulation at different frequencies and intensities in healthy humans and people with spinal cord injury. This approach will enable us to develop stimulation protocols that are optimal for recovery of standing and walking. Incorporation of computational models along with experimental data will further assist in defining the stimulation protocol that suits a specific patient and thus develop a real targeted treatment. A limitation of the study was that the control walking session (walking without transspinal stimulation) was always at the beginning of the data collection, which might result in an order effect—i.e., participants may perform better because of practice.

### Supplementary Information

Supplementary Figure S1.

## Data Availability

The raw data supporting the conclusions of this article will be made available by the authors, without undue reservation. Address all requests regarding data from this study to Dr. Andreas Skiadopoulos (Andreas.Skiadopoulos@csi.cuny.edu).

## Abbreviations

CPGs: Central pattern generators
CRP: Continuous relative phase
DFA: Detrended fluctuation analysis
DP: Deviation phase
EMG: Electromyographic
IC: Initial contact
IID: Independent and identically distributed
ISB: International Society of Biomechanics
ISw: Initial swing
LR: Loading response
LyE: Lyapunov exponent
m: Embedding dimension
MARP: Mean absolute relative phase
MOS: Margin of stability
MS: Midstance
MSw: Mid-swing
PSw: Pre-swing
SD: Standard deviation
SUB: Sub-threshold
SUP: Supra-threshold
TEP: Transspinal evoked potential
TS: Terminal stance
TSw: Terminal swing
α-DFA: Fractal scaling exponent from the detrended fluctuation analysis
τ: Appropriate delay time
