# Supplementary material for: Tapping into the human spinal locomotor centres with transspinal stimulation
Source: Sci Rep. 2024 Mar 12;14:5990. doi: 10.1038/s41598-024-56579-0 (PMC10933285; doi:10.1038/s41598-024-56579-0)
Supplement: Supplementary file 1 — Supplementary Figure S1. [file 41598_2024_56579_MOESM1_ESM.docx]

**
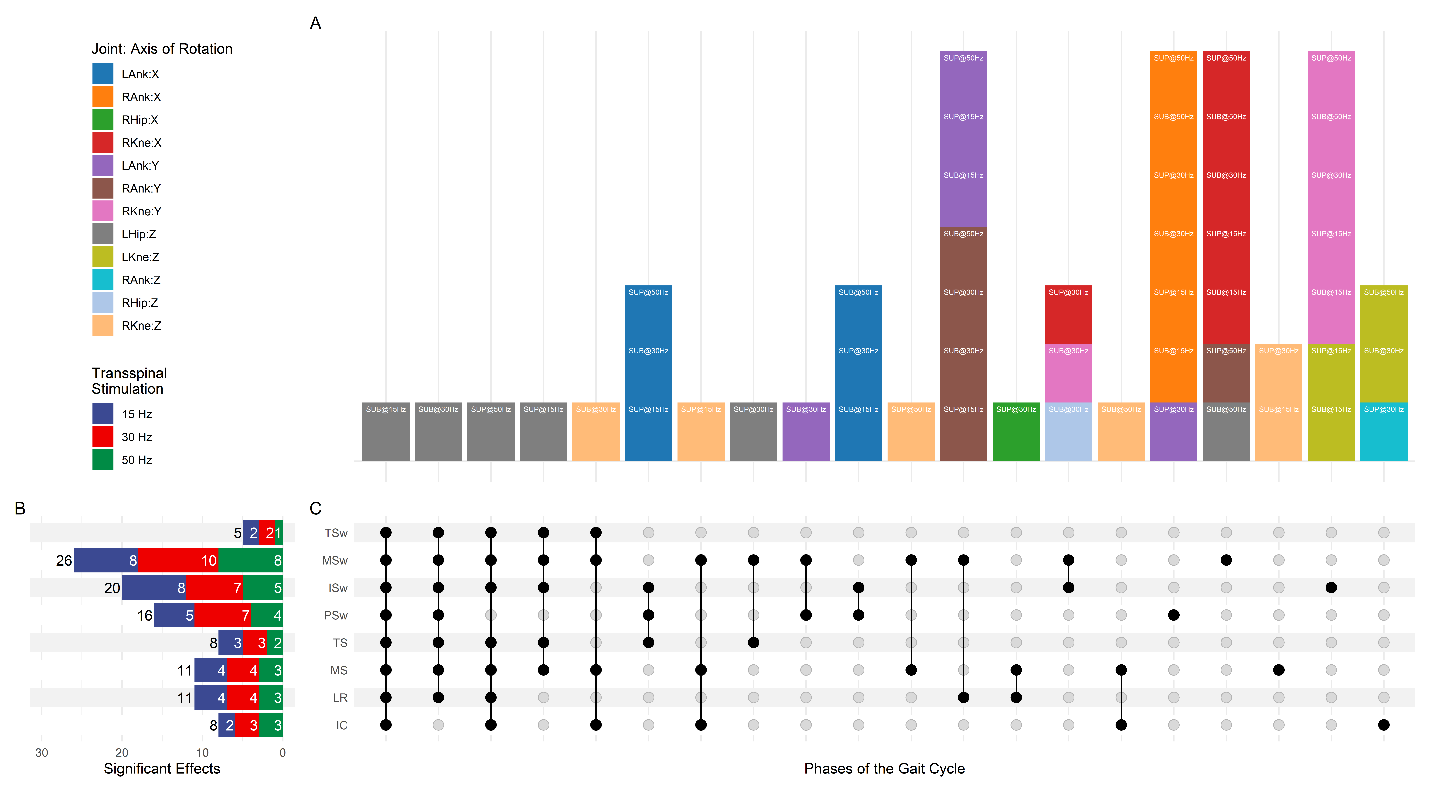
**

**Supplementary Figure 1.** Effects of transspinal stimulation on ankle, knee, and hip joint movements during walking. (A) Vertical bars display which gait phases affected by transspinal stimulation in relation to joint movements. (B) Horizontal stacked bars display the total number of significant effects of transspinal stimulation on joint movements for each gait phase and frequency. (C) Each row corresponds to a specific gait phase, and each column represents a distinct set of gait phases. Black dots signify gait phases in which transspinal stimulation had significant effect on joint movement compared to control walking. Multiple black dots in a column indicate significant transspinal stimulation effects across different gait phases. SUB = sub-threshold; SUP = supra-threshold; IC = initial contact; LR = loading response; MS = midstance; TS = terminal stance; PSw = pre-swing; ISw = initial swing; MSw = mid-swing; TSw = terminal swing; LAnk = Left Ankle; RAnk = Right Ankle; LKne = Left Knee; RKne = Right Knee; LHip = Left Hip; RHip = Right Hip; X = medio-lateral axis of rotation; Y = anteroposterior axis of rotation; Z = axial axis of rotation .
